# Quality Control of Protein Complex Assembly by a Transmembrane Recognition Factor

**DOI:** 10.1016/j.molcel.2019.10.003

**Published:** 2020-01-02

**Authors:** Nivedita Natarajan, Ombretta Foresti, Kim Wendrich, Alexander Stein, Pedro Carvalho

**Affiliations:** 1 Sir William Dunn School of Pathology, University of Oxford, South Parks Road, Oxford OX1 3RE, UK; 2 Cell and Developmental Biology Programme, Centre for Genomic Regulation (CRG), Dr. Aiguader, 88, 08003 Barcelona, Spain; 3 Research Group Membrane Protein Biochemistry, Max Planck Institute for Biophysical Chemistry, 37077 Göttingen, Germany

## Abstract

The inner nuclear membrane (INM) is continuous with the endoplasmic reticulum (ER) but harbors a distinctive proteome essential for nuclear functions. In yeast, the Asi1/Asi2/Asi3 ubiquitin ligase complex safeguards the INM proteome through the clearance of mislocalized ER membrane proteins. How the Asi complex selectively targets mislocalized proteins and coordinates its activity with other ER functions, such as protein biogenesis, is unclear. Here, we uncover a link between INM proteome identity and membrane protein complex assembly in the remaining ER. We show that lone proteins and complex subunits failing to assemble in the ER access the INM for Asi-mediated degradation. Substrates are recognized by direct binding of Asi2 to their transmembrane domains for subsequent ubiquitination by Asi1/Asi3 and membrane extraction. Our data suggest a model in which spatial segregation of membrane protein complex assembly and quality control improves assembly efficiency and reduces the levels of orphan subunits.

## Highlights

• Quality control of unassembled subunits of membrane complexes is restricted to the INM
• The Asi complex promotes degradation of folded but unassembled membrane proteins
• Binding of Asi2 to membrane domain of unassembled subunits mediates their recognition
• INM quality control maintains complex subunits within near-stoichiometric levels

## Graphical Abstract

**Figure:**
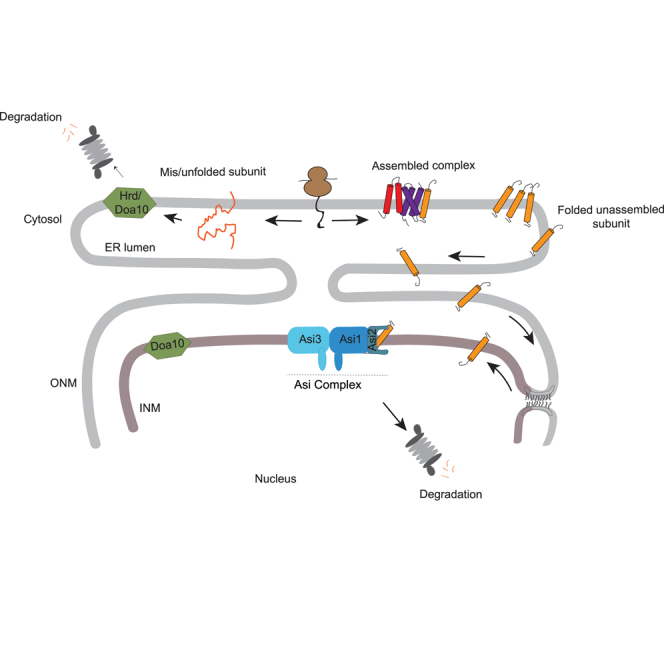

## Teaser

How subunits of membrane protein complexes are maintained at near-stoichiometric levels is unclear. Natarajan et al. show that assembly and quality control of membrane protein complexes are spatially uncoupled in yeast, with the degradation of unassembled subunits being restricted to the inner nuclear membrane and depending on the Asi ERAD complex.

## Introduction

The inner nuclear membrane (INM), which, together with the outer nuclear membrane, forms the nuclear envelope, is a specialized domain of the endoplasmic reticulum (ER). In contrast to bulk ER membranes that face the cytoplasm, the INM controls chromosome positioning within the nucleus, thereby influencing numerous processes from gene expression to DNA replication and repair (Hetzer, 2010, De Magistris and Antonin, 2018). These INM functions require a unique proteome that is distinct from that of the remaining ER membranes (Ungricht and Kutay, 2015). Mutations in INM proteins are frequently associated with diseases such as muscular dystrophies, progeroid syndromes, and cancer, underscoring the importance of maintaining protein homeostasis in this ER domain (Worman and Schirmer, 2015).

The INM is continuous with the remaining ER membrane, and its unique identity requires correct protein targeting. Upon synthesis and membrane insertion in the bulk ER, INM proteins diffuse in the membrane until they reach the INM, where they are retained through interactions with nuclear factors such as chromatin (Boni et al., 2015, Ungricht et al., 2015). Besides this diffusion-retention model, other mechanisms have been proposed for the targeting of proteins to the INM (Katta et al., 2014).

In yeast, the establishment of INM proteome identity is also achieved through the elimination of mislocalized proteins by ER-associated degradation (ERAD), a quality control process that includes multiple branches. Mislocalized proteins are targeted by an INM-specific ERAD branch defined by the Asi ubiquitin ligase complex (Foresti et al., 2014, Khmelinskii et al., 2014). Other ERAD branches encompass distinct ubiquitin ligase complexes, the Hrd1 and Doa10 complexes, which have major roles in the quality control of misfolded proteins in bulk ER membranes (Mehrtash and Hochstrasser, 2019, Ruggiano et al., 2014).

The Asi complex is composed of Asi1, Asi2, and Asi3; Asi1 and Asi3 contain RING domains, conferring ubiquitin ligase activity, while Asi2 does not have known functional domains. Mislocalized proteins ubiquitinated by the Asi complex are subsequently extracted from the INM by the soluble ATPase Cdc48 (p97 in mammals) in complex with its cofactors Npl4 and Ufd1 and handed to the proteasome for degradation (Bays et al., 2001, Foresti et al., 2014, Jarosch et al., 2002, Khmelinskii et al., 2014, Rabinovich et al., 2002, Ye et al., 2001). How the Asi complex specifically recognizes “mislocalized” proteins at the INM remains unclear. It is also unknown how the degradation of mislocalized proteins at the INM contributes to protein homeostasis in the bulk ER, as shown by previous genetic studies (Foresti et al., 2014, Khmelinskii et al., 2014).

Here, we uncover a link between INM proteome identity and quality control of the membrane protein complex assembly. Unassembled subunits of protein complexes constitute a significant burden to cells, as shown by recent proteomics experiments (McShane et al., 2016). However, quality control processes involved in their degradation have remained elusive (Juszkiewicz and Hegde, 2018). We show that folded unassembled subunits of protein complexes are not detected by ERAD in bulk ER membranes. Instead, these orphan subunits diffuse easily to the INM, where they are recognized by the Asi complex. Using *in vivo* crosslinking and *in vitro* reconstitution experiments, we show that recognition is mediated by the direct binding of Asi2 to substrate transmembrane domains (TMDs). Asi2 binding facilitates substrate ubiquitination and subsequent Cdc48-mediated extraction. We propose that restricting the quality control of unassembled proteins to the INM, a relatively small region of the ER that is not involved in protein biogenesis, spares subunits from premature degradation and offers them more time to find their partners. Thus, spatial segregation of the two processes, protein assembly (in the bulk ER) and quality control (at the INM), may facilitate efficient complex assembly.

## Results

### Asi Degrades Unassembled Complex Subunits

We previously showed that degradation of the Asi complex substrate Nsg1 was strongly accelerated in cells lacking its binding partner Hmg2 (Foresti et al., 2014). We also showed that Erg11, a p450 protein family member that noticeably does not assemble into stable complexes (Debose-Boyd, 2007, Hughes et al., 2007), was constitutively degraded in an Asi-dependent manner. These observations raise the possibility that by targeting lone or unassembled subunits, the Asi complex is involved in the quality control of protein complex assembly.

**Figure 1:**
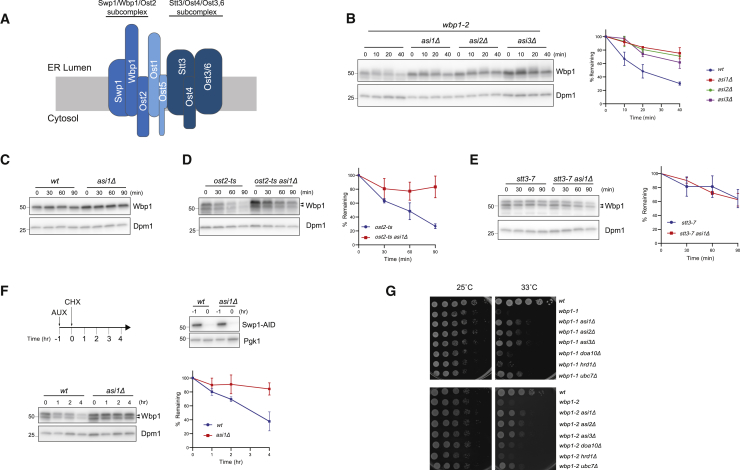
Orphan Subunits of ER Protein Complexes Are Degraded by the Asi Complex (A) Scheme of the OST complex. Different shades of blue indicate the three subcomplexes that form OST. (B) The degradation of the OST complex mutant subunit wbp1-2 was followed after inhibition of protein synthesis by cycloheximide (CHX) in cells with the indicated genotype upon a 45-min shift to 30°C. Wbp1 was detected with α-Wbp1 antibody. Dolichol phosphate mannose synthase (Dpm1) was used as loading control and detected with α-Dpm1 antibody. The graph (right) shows the quantification of at least three independent experiments; error bars represent the standard deviation. (C) The degradation of endogenous Wbp1 in WT and *asi1Δ* cells was analyzed as in (B). (D) The degradation of endogenous Wbp1 in cells with the indicated genotype was analyzed upon a 60-min shift to 37°C and samples processed as in (B). In *ost2-ts* cells, Wbp1 is hypoglycosylated and runs as a doublet (arrowheads). The graph (right) shows the quantification of at least three independent experiments; error bars represent the standard deviation. (E) The degradation of endogenous Wbp1 in cells with the indicated genotype was analyzed as in (D). In *stt3-7* cells, Wbp1 is hypoglycosylated and runs as a doublet (arrowheads). The graph (right) shows the quantification of at least three independent experiments; error bars represent the standard deviation. (F) The degradation of endogenous Wbp1 was followed after the inhibition of protein synthesis by CHX upon acute depletion of its binding partner Swp1-AID-FLAG (bottom). Dpm1 was used as loading control and detected with α-Dpm1 antibody. The graph (right) shows the quantification of at least three independent experiments; error bars represent the standard deviation. Auxin-induced Swp1-AID-FLAG depletion in WT and *asi1Δ* cells was confirmed by blotting with α-FLAG antibody (top). Pgk1 was used as loading control and detected with α-Pgk1 antibody. (G) Serial dilutions of cells with the indicated genotype were spotted on YPD and incubated for 2 days at 25°C and 33°C.

To test this hypothesis, we analyzed the oligosaccharyl transferase (OST; Figure 1A) and the glycosylphosphatidylinositol transamidase (GPI-T; Figure S1A) complexes, which are ER membrane protein complexes that are required for N-linked protein glycosylation and protein GPI anchor attachment, respectively (Benghezal et al., 1996, Fraering et al., 2001, te Heesen et al., 1993, Kelleher and Gilmore, 1994). Both OST and GPI-T complexes are essential for cell viability, and to conditionally perturb their assembly we took advantage of temperature-sensitive (ts) alleles of Wbp1, an OST subunit, and Gpi8, a GPI-T subunit. Mutant proteins encoded by ts alleles are commonly degraded at the restrictive temperature. We tested whether previously described ts alleles of Wbp1 and Gpi8 would result in unstable proteins (Ben-Aroya et al., 2008, te Heesen et al., 1993, Li et al., 2011). While both endogenous Wbp1 and Gpi8 are relatively long-lived proteins, their ts allele-encoded counterparts Wbp1-2 and Gpi8-ts were quickly degraded. In contrast, both were strongly stabilized in Asi mutants (Figures 1B, 1C, S1B, and S1C).

The assembly of the OST complex is well characterized (Kelleher and Gilmore, 1994), and its structure was recently solved (Bai et al., 2018, Wild et al., 2018). Moreover, the stability of the various subunits and their interdependencies are known (Mueller et al., 2015). Therefore, we considered it a good model to further study the role of the Asi complex in quality control of the protein complex assembly. The OST complex is composed of three subcomplexes (Figure 1A). One of these is formed by Wbp1, Ost2, and Swp1, with the stability of each subunit depending on the presence of the other two (Mueller et al., 2015). In cells expressing the *ost2-ts* allele, we observed rapid degradation of Wbp1. In contrast, the half-life of Wbp1 was extended in *ost2-ts* cells lacking Asi components (Figure 1D). Destabilization of the OST complex in the *ost2-ts* mutant was confirmed by immunoprecipitation experiments in cells expressing a functional tagged version of Ost4, another OST subunit (Figure S1E). In contrast, destabilization of Stt3, which belongs to a distinct OST subcomplex (Figure 1A) and does not interfere with the Wbp1-Swp1-Ost2 assembly (Mueller et al., 2015), did not affect Wbp1 levels and turnover (Figure 1E).

Furthermore, acute depletion of Swp1, also a binding partner of Wbp1, using an auxin-based degradation system (Morawska and Ulrich, 2013, Nishimura et al., 2009), resulted in the reduction of the half-life of the wild-type (WT) Wbp1 protein. Again, Wbp1 degradation was inhibited in Asi mutant cells (Figure 1F). These data indicate that unassembled subunits of protein complexes are targeted for degradation by the Asi complex at the INM.

We showed that under conditions of compromised assembly, Asi complex mutations lead to higher steady-state levels of protein complex subunits. However, whether these subunits would be competent for assembly and give rise to functional complexes was not clear. Thus, we tested whether Asi mutations could rescue the growth of ts alleles in OST and GPI-T complexes. The deletion of Asi components rescued the growth of both OST (*wbp1-1*, *wbp1-2*) and GPI-T (*gpi8-ts* and *gpi16-ts*) mutant alleles at the restrictive temperature, indicating that they gave rise to functional complexes (Figures 1G and S1D). The growth improvements were specific for the Asi complex mutations and were not observed in the mutants of other ERAD branches, including Hrd1 and Doa10. Thus, orphan subunits targeted by the Asi complex at the INM are folded and competent to assemble functional complexes. Furthermore, our data suggest that complex assembly in bulk ER membranes is facilitated by restricting quality control of the unassembled subunits to the INM.

### Erg11 TMD Is Sufficient for Its Degradation by Asi

**Figure 2:**
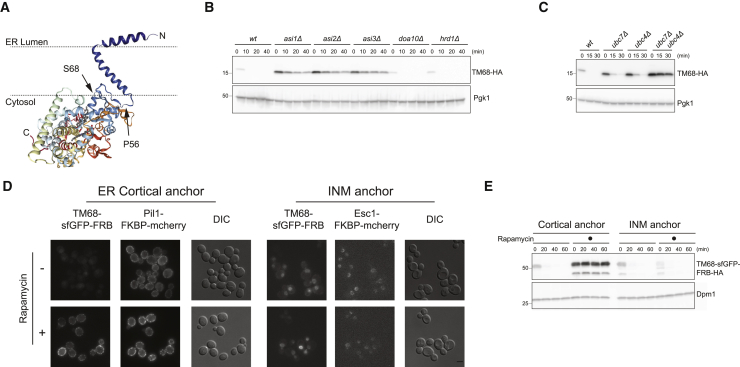
Erg11 TMD Is Sufficient for Its Asi-Mediated ERAD (A) Structure of full-length Erg11 according to Monk et al. (2014) and predicted orientation in the ER bilayer. Arrowheads indicate residues 56 and 68. (B) The degradation of plasmid-derived TM68-HA was followed after the inhibition of protein synthesis by CHX in cells with the indicated genotype. Whole-cell extracts were analyzed by immunoblotting. TM68 was detected with α-HA antibody. Pgk1 was used as loading control and detected with α-Pgk1 antibody. (C) The degradation of TM68-HA was analyzed as in (B) in cells with the indicated genotype. (D) Localization of TM68-sfGFP-FRB-HA in the absence or presence of the dimerization agent rapamycin in cells expressing the cortical (Pil1-FKBP-mCherry, left panel) or INM (Esc1-FKBP-mCherry, right panel) anchors. Scale bar, 2 μm. (E) The degradation TM68-sfGFP-FRB-HA was analyzed as in (B) in cells expressing cortical (Pil1-FKBP-mCherry) or INM (Esc1-FKBP-mCherry) anchors in the absence or presence of the dimerization agent rapamycin.

To further investigate the mechanisms by which the Asi complex recognizes its substrates, we focused on the lone protein Erg11, a constitutive and robust Asi substrate (Foresti et al., 2014, Khmelinskii et al., 2014). Erg11 is anchored at the ER through a single N-terminal TMD, while the extended cytoplasmic C terminus consists of a cytochrome p450 domain that is essential for ergosterol biosynthesis (Figure 2A) (Monk et al., 2014).

To identify the minimal region of Erg11 involved in Asi-dependent degradation, we used truncation analysis. A small N-terminal region encompassing an ER luminal amphipathic helix (residues 6–23) followed by the TMD αhelix (27–51) was sufficient for Asi-dependent degradation. Like full-length Erg11 (Foresti et al., 2014), derivatives including only the first 56 or 68 amino acids followed by a hemagglutinin (HA) epitope, hereafter called TM56 and TM68 (Figure S2A), still associate with the ER membrane (Figure S2B). TM56 and TM68 were quickly degraded in WT cells as well as in *hrd1Δ* and *doa10Δ* ERAD mutants. In contrast, degradation of TM56 and TM68 was severely delayed in cells lacking any of the Asi complex components Asi1, Asi2, or Asi3 (Figures 2B and S2C). We previously showed that the ubiquitin-conjugating enzymes Ubc4 and Ubc7 assist the Asi complex in substrate degradation (Foresti et al., 2014). Similarly, we found that while individual mutations in Ubc4 and Ubc7 delayed the turnover of truncated Erg11 derivatives, their simultaneous deletion prevented the degradation of both TM56 and TM68 (Figures 2C and S2D).

Degradation of Asi substrates is restricted to the INM (Foresti et al., 2014). However, given the extremely short half-lives of TM56 and TM68 compared to full-length Erg11, we wanted to confirm that INM localization was a prerequisite for their degradation. To this end, we manipulated TM68 distribution within the ER by exploiting the conditional dimerization of FRB and FK506-binding protein (FKBP) domains induced by the small molecule rapamycin (Haruki et al., 2008, Spencer et al., 1993). TM68 was fused to the GFP and FRB domain (TM68-GFP-FRB), a construct that was still degraded in an Asi-dependent manner (Figure S2E). We coexpressed TM68-GFP-FRB with fusions of FKBP and monomeric cherry fluorescent protein to either Pil1 (Pil1-FKBP-mCherry), a protein stably associated with the cell cortex (Gallego et al., 2013, Ziółkowska et al., 2011), or Esc1 (Esc1-FKBP-mCherry), a protein stably associated with the INM (Andrulis et al., 2002). In the absence of the dimerization-inducing agent rapamycin, TM68-GFP-FRB was quickly degraded, irrespective of the coexpressed FKBP fusion. Likewise, in cells expressing the INM localized Esc1-FKBP-mCherry, the addition of rapamycin did not affect the turnover of TM68-GFP-FRB (Figure 2E). In contrast, the addition of rapamycin to cells expressing cortically associated Pil1-FKBP-mCherry induced the trapping of TM68-GFP-FRB in the peripheral ER (Figure 2D), resulting in its complete stabilization (Figure 2E). Thus, like other Asi substrates, degradation of TM68-GFP-FRB requires its diffusion to the INM. The fact that TM68-GFP-FRB was quick and efficiently trapped at the INM or peripheral ER indicates that it rapidly explores the entire ER membrane. These data show that the degradation of TM56 and TM68 has the same genetic and spatial requirements of their full-length counterpart Erg11.

### TMDs Act as Asi-Dependent Degrons

**Figure 3:**
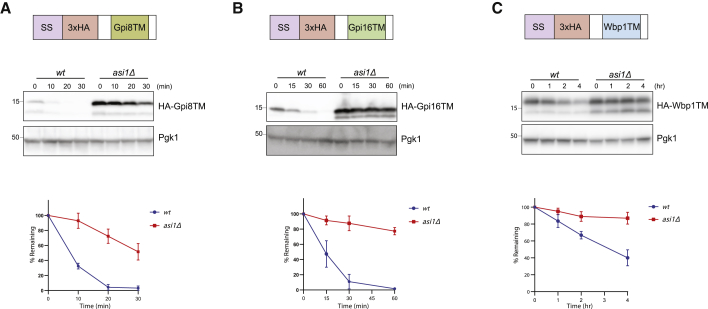
TMDs Act as Asi-Dependent Degrons (A) The degradation of HA-Gpi8 TM was analyzed as in Figure 2B. Schematic representation of HA-Gpi8TM is shown (top) with the various modules of the construct: a signal sequence (SS) followed by a 3xHA tag and the TM domain. The graph (bottom) shows the quantification of at least three independent experiments; error bars represent the standard deviation. (B) The degradation of HA-Gpi16 TM domain was analyzed as in (A) in WT and *asi1Δ* cells. (C) The degradation of HA-Wbp1 TM domain was analyzed as in (A) in WT and *asi1Δ* cells.

To test whether other TMDs also function as Asi degradation signals, we generated constructs with the TMDs of the ER proteins Wbp1 and Gpi8 and Gpi16 belonging, respectively, to the OST and GPI-T complexes described earlier. These constructs also included an N-terminal signal sequence for ER targeting followed by a HA epitope tag. In WT cells, these TMDs were unstable and degraded with varying half-lives (Figures 3A–3C). However, TMD degradation was strongly delayed by Asi complex mutations, while mutations in other ERAD complexes had a much weaker effect (Figures S3A–S3C). These data show that distinct TMDs encompassing a range of biophysical properties (Figure S3D) are degraded in an Asi-dependent manner. Thus, it seems that TMD α helices define a degradation signal for the Asi complex at the INM.

### Asi Components Crosslink to TMD Substrates

To investigate interactions of TMD degrons with the Asi complex, we used a sensitive *in vivo* site-specific photocrosslinking approach (Chin et al., 2003). Using a similar strategy, we previously characterized the interactions between ERAD luminal substrates and components of the Hrd1 complex (Carvalho et al., 2010, Stanley et al., 2011). This system exploits an amber codon suppressor tRNA and a modified tRNA synthetase to introduce a photoreactive amino acid derivative (benzoyl-phenylalanine [Bpa]) at sites specified by an amber stop codon. In cells expressing this system and grown in the presence of Bpa, UV irradiation triggers crosslinks of the Bpa-labeled probes to proteins in close proximity (Chin et al., 2003).

**Figure 4:**
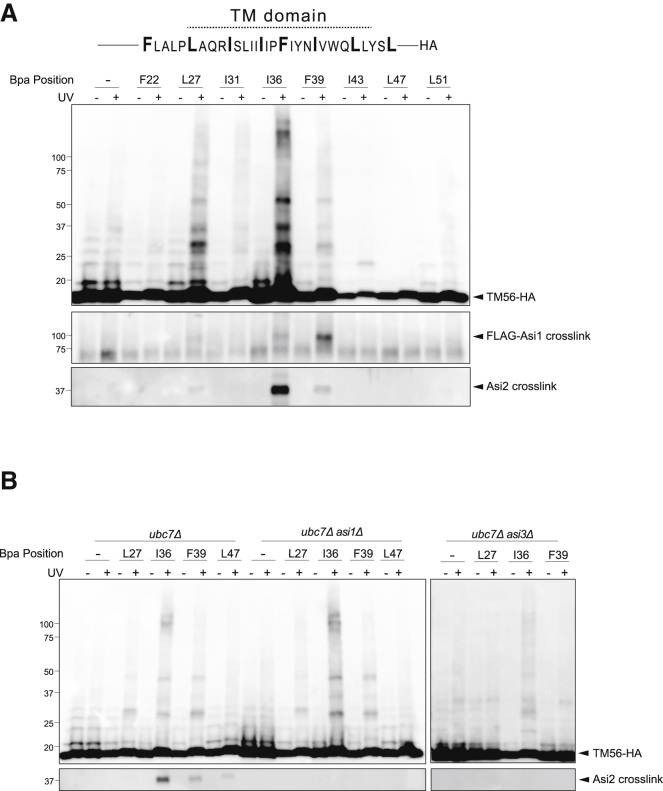
Asi Complex Crosslinks to Transmembrane Substrates (A) *ubc7Δ* cells with chromosomally tagged FLAG-Asi1 expressed from the *ADH1* promoter and plasmid-borne TM56 with the photoreactive amino acid analog benzoyl-phenylalanine (Bpa) at the indicated positions were subjected to UV irradiation. Non-irradiated cells were used as controls. Solubilized membranes were subjected to immunoprecipitation with anti-HA antibodies, and bound proteins were analyzed by immunoblotting with HA, FLAG, and Asi2 antibodies. (B) Cells of the indicated genotype and expressing TM56 derivatives with Bpa at selected positions were analyzed as in (A).

We generated TM56 derivatives with a single photoreactive Bpa label at various positions (four to five residues apart) throughout the TMD (Figure 4A). Given the low steady-state levels and short half-life of TM56 in WT cells (Figure S2C), crosslinking experiments were performed in *ubc7Δ* mutants in which TM56 levels are higher (Figure S2D). Cells expressing the various Bpa-labeled TM56 derivatives were UV irradiated, crude membrane extracts were prepared, and TM56 derivatives were immunoisolated and analyzed by SDS-PAGE followed by immunoblotting (Figure 4A). Cells expressing unlabeled TM56 or non-irradiated cells showed only minor unspecific bands. In contrast, UV-dependent crosslinks were observed for several Bpa positions. The pattern of crosslinks was similar for various positions (for example, 27, 31, 36, 39) (Figure 4A). The identity of the most prominent UV-dependent crosslinks remains unknown, but these crosslinks were independent of a functional Asi complex (Figure 4B).

To test whether Asi complex components were among the TM56 crosslinking partners, we used immunoblotting. We found that both FLAG-Asi1 and Asi2 crosslinked robustly with Bpa probes at positions 36 and 39 (Figure 4A). Crosslinks were also observed at neighboring positions (27 and 47) but became weaker as the Bpa probe was moved away from membrane equatorial positions. Crosslinks between TM56 and Asi2 required a full Asi complex and were lost in cells lacking Asi1 or Asi3 (Figure 4B). The loss of Asi2 interactions was not due to changes in its expression level, which in the mutants was similar to WT cells (Figure S4A). Finally, similar crosslinks between Asi2 and TM56 were observed in WT cells; however, these were much weaker due to the lower TM56 levels (Figure S4B). These data show that Asi complex components directly interact with a substrate’s TMD.

### *In Vitro* Reconstitution of Asi-Mediated ERAD

Previous studies implicated the ERAD complexes in the recognition, ubiquitination, and retrotranslocation of substrates (Baldridge and Rapoport, 2016, Carvalho et al., 2010, Christianson et al., 2011, Denic et al., 2006, Gauss et al., 2006, Stein et al., 2014). To test whether the Asi complex defined the minimal unit necessary for the recognition, ubiquitination, and retrotranslocation of its membrane-bound substrates, we developed an *in vitro* system recapitulating these ERAD steps. Asi1, Asi2, and Asi3 were purified as a complex from *Saccharomyces cerevisiae* either through a streptavidin-binding peptide tag fused to Asi2 (SBP-Asi2) or through FLAG tag on Asi3 (FLAG-Asi3) (Figure S5A). Both fusion proteins were functional (Figure S5B) (Foresti et al., 2014). In parallel, we recombinantly expressed and purified the ubiquitin-activating enzyme Uba1, the ubiquitin-conjugating enzymes Ubc4 and Ubc7, and its activator Cue1, all required for the ubiquitination reaction (Figure S5C). Recombinantly expressed TM68 fused to the maltose-binding protein (TM68-MBP) was used as the substrate. TM68-MBP also included a sortase recognition peptide, which allowed its fluorescent labeling for easy detection (Figure S5C). Ubiquitination experiments in the mild detergent decyl maltose neopentyl glycol (DMNG), which preserved Asi complex integrity, led to very minor TM68-MBP modification, which could be detected only after the enrichment of ubiquitin conjugates (Figure S5D). The very low modification of TM68-MBP was not due to the decreased activity of the purified Asi complex in detergent, as prominent ubiquitination of Asi components was detected in the same reactions (data not shown).

**Figure 5:**
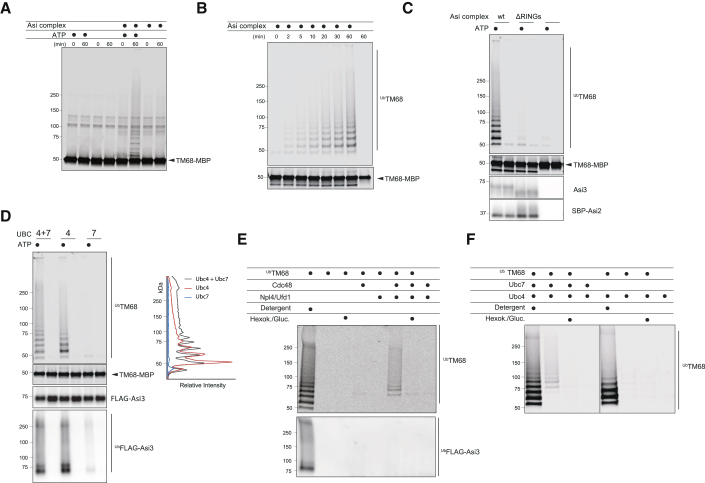
*In Vitro* Reconstitution of Asi-Mediated ERAD (A) Proteoliposomes containing fluorescently labeled TM68-MBP alone or together with the Asi complex were incubated with soluble ubiquitination machinery (Ubi Mix) during the indicated time in the presence or absence of ATP. Samples were resolved by SDS-PAGE and analyzed by fluorescence scanning. (B) Proteoliposomes containing Asi complex were incubated with Ubi Mix and ATP for the indicated times. Reactions were divided in two, with one part analyzed by fluorescence scanning (bottom panel; TM68-MBP) and the other subjected to His-ubiquitin affinity purification. Eluted proteins were analyzed by fluorescence scanning (top panel; ^Ub^TM68). (C) Proteoliposomes containing either WT or RING-deficient Asi complex were incubated with Ubi Mix for 60 min in the presence or absence of ATP. Proteoliposomes lacking Asi complex were used as control. Reactions were either analyzed directly by immunoblotting with α-SBP and α-Asi3 antibodies or fluorescence scanning (TM68-MBP) (bottom panels) or subjected to His-ubiquitin affinity purification. Eluted proteins were analyzed by fluorescence scanning (top panel; ^Ub^TM68). (D) Proteoliposomes containing the Asi complex were incubated with ubiquitin-activating enzyme, ubiquitin, the indicated ubiquitin-conjugating enzymes, and ATP for 60 min. Reactions were analyzed directly by SDS-PAGE (center panels) or subjected to His-ubiquitin affinity purification. Eluted proteins were analyzed by fluorescence scanning (top panel) or immunoblotting (bottom panel). Line scan fluorescence intensity profiles are graphed at right. (E) Proteoliposomes containing fluorescently labeled TM68 and the Asi complex were immobilized on streptavidin magnetic beads and subjected to ubiquitination reactions for 60 min. Substrate extraction was initiated by the addition of the indicated Cdc48 ATPase complex components in the presence or absence of hexokinase/glucose for ATP depletion and incubated for 30 min. Extracted material was recovered by His-ubiquitin affinity purification. Eluted proteins were analyzed by fluorescence scanning (TM68-MBP) or immunoblotting (FLAG-Asi3). (F) Proteoliposomes containing fluorescently labeled TM68-MBP and the Asi complex were immobilized on streptavidin magnetic beads and subjected to ubiquitination with the indicated ubiquitin conjugating enzymes. Substrate extraction reactions were performed as in (E).

We hypothesized that the detergent micelle did not adequately recapitulate the environment of a phospholipid bilayer, considering that Asi-substrate interaction occurs within the membrane. We therefore co-reconstituted the Asi complex and TM68-MBP into liposomes. Floatation and pull-down experiments confirmed co-reconstitution of the various membrane components (Figures S5E and S6A). In proteoliposomes, the majority of both TM68-MBP substrate and the Asi complex adopted a topology with their cytoplasmic domains facing the outside, as assayed by protease protection (Figure S5F). Proteoliposomes containing Asi complex and TM68-MBP were incubated with the soluble ubiquitination machinery (Ubi Mix: Uba1, Ubc4, Ubc7, Cue1, ubiquitin) in either the presence or the absence of ATP. Reactions were analyzed by SDS-PAGE and TM68-MBP visualized by fluorescence. TM68-MBP was readily detected in high-molecular-weight bands strongly suggestive of ubiquitination (Figure 5A). Consistently, the presence of slow migrating TM68-MBP species required the presence of ATP and the Asi complex. To confirm that the high-molecular-weight bands correspond to ubiquitinated TM68-MBP, ubiquitin conjugates were precipitated under denaturing conditions and eluted material was analyzed as above. Over time, a fraction of TM68-MBP was ubiquitinated (Figure 5B). TM68-MBP ubiquitination was dependent on Asi complex ubiquitin ligase activity, as TM68-MBP was not modified in experiments carried out with ubiquitination-deficient Asi complex (Figure 5C). *In vitro*, the specificity of ubiquitin ligases is often reduced, resulting in the ubiquitination of any protein in close proximity. To assess the specificity of the Asi complex *in vitro*, we performed ubiquitination reactions with TMDs of two INM resident proteins that are not degraded by Asi *in vivo*, Mps3 and Ubc6. Mps3 was recently shown to be degraded by the soluble E3 ubiquitin ligase anaphase-promoting complex (APC)/Cdh1 (Koch et al., 2019), while Ubc6 is a well-established substrate of the E3 ligase Doa10 (Swanson et al., 2001). The TMDs of Mps3 and Ubc6 are much less efficiently ubiquitinated by the Asi complex than TM68-MBP (Figure S5G). In the case of Ubc6, Asi-dependent ubiquitination increases at higher substrate concentrations, while Mps3 modification remains low, even when it is present at high concentrations in the proteoliposomes (Figure S5G). Liposome floatation assays show that the differences in ubiquitination do not result from diminished protein reconstitution efficiency (Figure S5H). Thus, the Asi complex is necessary and sufficient for the recognition and ubiquitination of integral membrane substrates.

In cells, the Asi-mediated ERAD branch depends on the ubiquitin-conjugating enzymes Ubc4 and Ubc7 (Foresti et al., 2014). To test whether there was a similar dependence *in vitro* and the individual contribution of the conjugating enzymes, we performed reactions with only Ubc4 or Ubc7. With only Ubc4, TM68-MBP was still ubiquitinated, but the conjugates had a distinct profile, with an increase in monoubiquitinated species and a reduction in high-molecular-weight conjugates, when compared to reactions with both Ubc4 and Ubc7 (Figure 5D). Asi3 was also ubiquitinated *in vitro*, a modification mostly dependent on Ubc4 (Figure 5D). Substrate ubiquitination was absent in reactions containing exclusively Ubc7 (Figure 5D), even if this enzyme was active, as confirmed by *in vitro* reactions with the ubiquitin ligase Hrd1 (data not shown). These results suggest that Ubc4 initiates the ubiquitination of Asi substrates. In constrast, Ubc7 appears unable to initiate substrate ubiquitination but efficiently extends ubiquitin chains.

Finally, we tested whether ubiquitinated TM68-MBP could be retrotranslocated and extracted from the proteoliposomes. To this end, ubiquitination was performed in proteoliposomes immobilized onto streptavidin beads using SBP-Asi2 and substrate extraction was initiated by the addition of the Cdc48/Npl4/Ufd1 ATPase complex. Extraction of TM68-MBP required its ubiquitination, the presence of a complete Cdc48 ATPase complex, and ATP (Figure 5E). Cdc48-dependent extraction appears substrate specific, as we could not detect the extraction of polyubiquitinated Asi3 molecules (Figure 5E). Only substrate carrying more than three to four ubiquitin molecules was efficiently extracted from proteoliposomes, suggesting a ubiquitin-chain size dependence for Cdc48 activity. Considering the effects of Ubc4 and Ubc7 in Asi-dependent substrate ubiquitination, we tested whether their concerted activity was necessary for substrate extraction. Cdc48 was unable to extract substrate molecules ubiquitinated in the presence of Ubc4 alone, in contrast to reactions containing both Ubc4 and Ubc7 (Figure 5F). These data suggest that Ubc4 is competent for monoubiquitination of multiple lysine residues but unable to generate polyubiquitin chains, shown to preferentially bind to the Cdc48 ATPase complex (Bodnar and Rapoport, 2017). Our data show that the Asi complex defines the minimal unit required for ERAD of INM substrates.

### Asi2 Is a Transmembrane Recognition Factor

**Figure 6:**
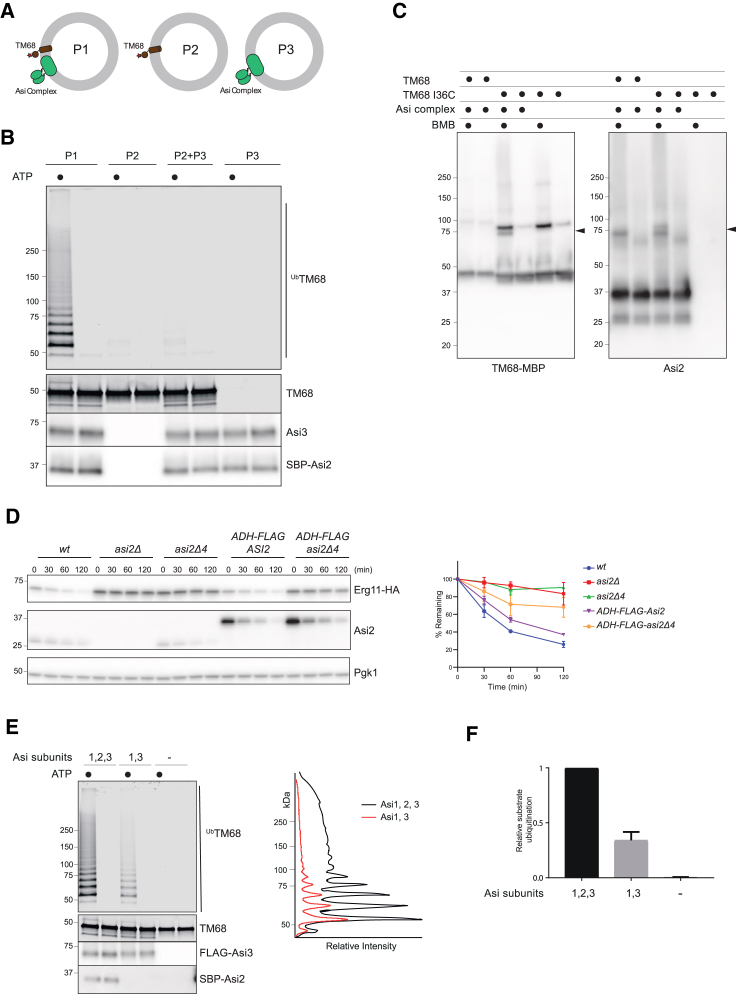
Asi2 Is a Transmembrane Recognition Factor (A) Schematic representation of the proteoliposomes used in (B). (B) The indicated proteoliposomes were incubated with Ubi Mix for 60 min in the presence or absence of ATP. Reactions were either analyzed directly by immunoblotting (bottom panels) or subjected to His-ubiquitin affinity purification. Eluted proteins were analyzed by fluorescence scanning (TM68). (C) Proteoliposomes containing Asi complex either with TM68-MBP or a derivative with a cysteine residue at position 36 (I36C-MBP) were incubated with the bifunctional cysteine-reactive crosslinker BMB, as indicated. Reactions were analyzed by immunoblotting with antibodies against MBP (left panel) and Asi2 (right panel). (D) The degradation of Erg11-HA was analyzed as in Figure 2B. Erg11 and Asi2 were detected with α-HA and α-Asi2 antibodies, respectively. The graph shows the quantification of at least three independent experiments; error bars represent the standard deviation. (E) Proteoliposomes containing either the Asi1/Asi2/Asi3 complex or the Asi1/Asi3 subcomplex were incubated with Ubi Mix for 60 min in the presence or absence of ATP. Proteoliposomes lacking the Asi complex were used as controls. Reactions were analyzed directly by immunoblotting (bottom panels) or subjected to His-ubiquitin affinity purification. Eluted proteins were analyzed by fluorescence scanning (TM68-MBP). Line scan fluorescence intensity profiles are graphed on the right. (F) Quantification of TM68 ubiquitination from reactions performed as in (E). Three ubiquitination reactions from independent reconstitutions experiments were quantified; error bars represent the standard deviation.

Our *in vivo* crosslinking experiments suggest that the Asi ubiquitin-ligase complex is involved in recognizing its substrates within the membrane. To directly test whether interactions with the TMD are a prerequisite for substrate recognition and ubiquitination, we took advantage of the described *in vitro* system. TM68-MBP and the Asi complex were either reconstituted together in the same proteoliposomes or individually into different proteoliposomes (Figure 6A). Ubiquitination reactions were performed as before using the individual proteoliposomes or by mixing proteoliposomes containing either the Asi complex or TM68-MBP. Substrate ubiquitination was detected only if the substrate and the Asi complex were in the same proteoliposome (Figures 6B and S6A), further indicating that substrate recognition, preceding ubiquitination, occurs through the TMD.

To gain more insight into the mechanism of substrate recognition, we set out to identify its main interactor(s) within the Asi complex. To this end, a version of TM68-MBP containing a single cysteine within its TMD (at position 36) was recombinantly expressed, purified, and co-reconstituted with the Asi complex into proteoliposomes. Single cysteine TM68(I36C)-MBP behaved indistinguishably from TM68-MBP (Figure S6B). To identify TM68(I36C)-MBP binding partners within the membrane, proteoliposomes were treated with the bifunctional sulfhydryl crosslinker 1,4-bismaleimidobutane (BMB). Proteoliposomes containing either only TM68-MBP or lacking the Asi complex were used as controls. After quenching, crosslinking reactions were analyzed by SDS-PAGE and immunoblotting. Besides a TM68(I36C)-MBP dimer, we detected a crosslinked product of ∼75 kDa. This crosslink was observed exclusively in samples treated with BMB and only if TM68(I36C)-MBP and the Asi complex were included (Figure 6C). Considering the molecular weight of Asi complex components, the 75-kDa band could correspond to a crosslink between TM68(I36C)-MBP and SBP-Asi2, which is ∼37 kDa. This was confirmed by analyzing the crosslinking reactions with an anti-Asi2 antibody (Figure 6C). Within the Asi complex, Asi2 has the smallest number of cysteines (only 1 versus 19 in Asi1 and 15 in Asi3), further supporting the specificity of the crosslink. Moreover, these data are consistent with the *in vivo* site-specific photocrosslinking experiments in which prominent crosslinks between TM56 and Asi2 were detected (Figure 4). Thus, among Asi complex components, Asi2 is the main interactor with a TMD substrate.

The single cysteine in Asi2 lies on a predicted TMD proximal to its C terminus. Our crosslinking data show that this TMD is in close proximity to substrates, and we wondered whether it would be necessary for substrate recognition and degradation. To test this hypothesis, we deleted four amino acids (residues 261–264, corresponding to Leu, Cys, Leu, and Leu, respectively) to generate a mutant Asi2 protein, Asi2Δ4, with a shortened TMD. In cells, Asi2Δ4 assembled into the Asi complex (Figure S6C) and was expressed at near-normal levels (Figures 6D and S6C); however, Asi2Δ4 was unable to promote the efficient degradation of Asi substrate Erg11, even if overexpressed (Figure 6D). This indicates that Asi2-substrate interactions within the membrane are required for substrate recognition and efficient degradation.

Finally, we tested the role of Asi2 in substrate recognition using the *in vitro* assay described above. Proteoliposomes containing TM68-MBP and the entire Asi complex or only the Asi1/Asi3 subcomplex were used in the ubiquitination reactions. In the absence of Asi2, substrate ubiquitination was reduced to 28% (±8%) (Figure 6E). This was not a consequence of a general decrease in Asi1/Asi3 ubiquitin ligase activity, as Asi3 was comparably ubiquitinated irrespective of the presence of Asi2 (Figure 6E; data not shown). Thus, Asi2-mediated recognition facilitates substrate ubiquitination and degradation both *in vitro* and *in vivo*.

## Discussion

Here, we used genetic, biochemical, and *in vitro* reconstitution approaches to characterize the mechanism of INM quality control by the Asi complex. We found an unexpected link between the INM proteome identity and quality control of the protein complex assembly.

**Figure 7:**
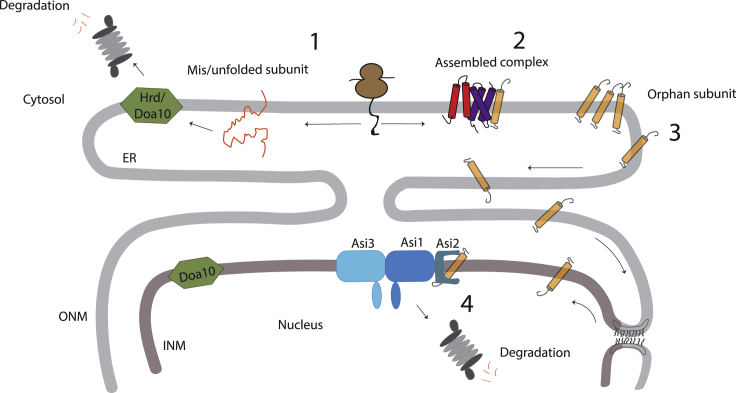
Working Model for Asi-Mediated Quality Control of the Protein Complex Assembly Schematic representation of biogenesis and quality control of membrane protein complexes in the ER. Complex subunits that misfold are degraded by the Hrd1 or Doa10 ERAD branches in bulk ER membranes (gray). Subunits that fold but fail to assemble eventually diffuse into the INM (brown), where they are degraded in Asi-dependent ERAD.

Based on our findings, we propose the following stepwise model for the quality control of the protein complex assembly (Figure 7). Newly synthesized subunits that misfold are quickly targeted for degradation by ERAD complexes in the bulk ER, such as Hrd1 and Doa10 (1). Subunits that fold but do not assemble immediately can diffuse through the ER membrane. This provides an opportunity to bind to their partners and for successful complex assembly (2). Compared to unassembled subunits, assembled complexes have an increased number of TMDs and often larger cytoplasmic domains, factors that hinder their passage through the pore membrane to the INM (Ungricht and Kutay, 2015). Moreover, assembled complexes likely interact with cytosolic components, which also contribute to their retention in the ER membranes exposed to the cytosol. Subunits that fail to find their partners or are present in over-stoichiometric amounts will persistently diffuse through the ER and eventually reach the INM (3), where the Asi complex promotes their degradation (4). Thus, we postulate that in yeast, bulk ER membranes and INM define areas favoring the assembly and degradation of folded, unassembled complex subunits, respectively. Whether a similar spatial segregation between complex assembly and quality control also occurs in mammalian cells should be investigated. In mammalian cells, the degradation of proteins directly from the INM by an ERAD-like process was described for a mutant version of the lamin-B receptor (Tsai et al., 2016), but the components involved in the degradation have not been identified.

Our findings are in agreement with earlier studies on the OST complex assembly in yeast that showed a very minor contribution of Hrd1 and Doa10 ERAD branches in the degradation of unassembled subunits and only if these were overexpressed, a condition that likely favors protein misfolding (Mueller et al., 2015).

The model proposed here is also consistent with recent proteomics studies on age-dependent protein degradation. These showed that many subunits of protein complexes display a biphasic degradation kinetics, being short-lived immediately after synthesis and prior to assembly, but becoming more stable as they age and after successful assembly with their partners (McShane et al., 2016).

Genetic suppression of ts alleles in Asi complex mutants argues for a major role of this ERAD branch in the degradation of orphan subunits at the INM. While the analysis focused on a limited number of complexes, our conclusions are further supported by unbiased large-scale genetic interaction studies (Van Leeuwen et al., 2016), in which Asi mutants suppress conditional alleles on the subunits of additional complexes. However, we cannot exclude that other ERAD branches may contribute to quality control of the complex assembly. The Doa10 complex also localizes to the INM and has nuclear substrates such as the soluble transcriptional repressor Mat2α (Deng and Hochstrasser, 2006, Swanson et al., 2001) and the membrane proteins Asi2 (Boban et al., 2014) and Ubc6 (Walter et al., 2001). However, it is unclear whether the degradation of these substrates depends on their assembly state; in its orphan state, the translocon subunit Sbh2 is degraded in a Doa10-dependent manner (Boban et al., 2014, Habeck et al., 2015). Curiously, Sbh2, but not its paralog Sbh1, was detected at the INM (Smoyer et al., 2016), but whether the INM pool of Sbh2 is the one targeted by Doa10 is unknown.

We showed that Asi2 directly recognizes lone and orphan proteins. Recognition requires substrate INM localization and is mediated by Asi2 binding to TMDs. While all of the substrates analyzed here contain a single TMD, other Asi2 substrates are multispanning membrane proteins (Foresti et al., 2014, Khmelinskii et al., 2014). Thus, Asi2 likely also recognizes TMDs within multipass proteins. The recognition of substrates based on their orphan state is common to other factors involved in the quality control of mislocalized membrane proteins. This is the case for the outer mitochondrial ATPase Msp1 (Weir et al., 2017), involved in quality control of mislocalized tail-anchored proteins (Chen et al., 2014, Okreglak and Walter, 2014), and the retrieval factor Rer1, which transports unassembled membrane proteins from the Golgi back to the ER (Sato et al., 2001, Sato et al., 2003). Thus, the absence of a binding partner may be a general feature used in the quality control of mislocalized membrane proteins.

The identification of structural features recognized by Asi2 on TMDs will require analysis of a larger number of substrates. However, the diverse properties of TMDs tested here (Figure S3) suggest that Asi2 has broad specificity. Nevertheless, among the Asi substrates identified so far there are no INM resident proteins, suggesting that additional regulation spares these proteins from Asi-dependent degradation (Foresti et al., 2014, Khmelinskii et al., 2014, Smoyer et al., 2019).

The degradation of certain Asi substrates does not require Asi2 (Foresti et al., 2014, Khmelinskii et al., 2014). Whether Asi2-independent substrates are directly recognized by Asi1 and Asi3 or involve additional unknown factors is not clear yet. However, the existence of multiple substrate recognition modules within the same ERAD complex is common (Christianson et al., 2011, Kanehara et al., 2010).

We showed that degradation of Asi substrates involved the ubiquitin conjugating enzymes Ubc7 and Ubc4 (Foresti et al., 2014). *In vitro* Ubc4 and Ubc7 conjugating enzymes are required for distinct, non-redundant functions in substrate ubiquitination. Ubc4 is required to conjugate initial ubiquitin molecules on the substrate, which are subsequently extended into polyubiquitin chains by Ubc7. A similar priming followed by Ubc7-dependent extension was observed for Doa10-dependent ubiquitination. In this case, priming was mediated by the conjugating enzyme Ubc6 and was particularly important for the ubiquitination of lysine-poor substrates, as Ubc6 facilitates ubiquitin conjugation to lysine and hydroxylated residues (Weber et al., 2016).

Our *in vitro* system faithfully recapitulated Cdc48-dependent membrane extraction of ubiquitinated substrates. Only substrates ubiquitinated in the presence of Ubc4 and Ubc7 conjugating enzymes and having four or more ubiquitins were extracted by Cdc48. This is in agreement with Cdc48 ubiquitin-binding preferences (Bodnar and Rapoport, 2017). Asi3 was also prominently ubiquitinated *in vitro*, but whether this is physiological, as in the case of Hrd1 (Baldridge and Rapoport, 2016), or a side reaction of the system is unclear. Ubiquitinated Asi3 did not appear to be extracted from proteoliposomes. Whether Cdc48 discriminates substrate and Asi3 based on different ubiquitin linkages or by some other mechanism should be clarified. It has been shown that Hrd1 ubiquitin ligase facilitates the retrotranslocation and membrane extraction of its substrates (Baldridge and Rapoport, 2016, Carvalho et al., 2010, Schoebel et al., 2017), but whether other ERAD ligases, such as Asi, work in a similar fashion should also be the focus of future studies.

## STAR★Methods

### Key Resources Table

**Table:** 

REAGENT or RESOURCE	SOURCE	IDENTIFIER
**Antibodies**		

Rat monoclonal anti-HA	Roche	11867431001
Mouse monoclonal anti-Pgk1	Invitrogen	459250
Mouse monoclonal anti-Dpm1	Invitrogen	A-6429
Mouse monoclonal anti-FLAG M2-Peroxidase (HRP)	Sigma-Aldrich	A8592
Mouse monoclonal anti-FLAG M2	Sigma-Aldrich	F1804
Mouse monoclonal anti-SBP tag, clone 20	Merck	MAB10764
Mouse monoclonal anti-MBP	New England BioLabs	E8032S
Rabbit polyclonal anti-Wbp1	H. Riezman lab	N/A
Rabbit polyclonal anti-Gpi8	A. Conzelmann lab	N/A
Rabbit polyclonal anti-Asi1	This study	N/A
Rabbit polyclonal anti-Asi2	This study	N/A
Rabbit polyclonal anti-Asi3	This study	N/A

**Bacterial and Virus Strains**		

BL21-CodonPlus (DE3)-RIPL cells	230280	Agilent Technologies

**Chemicals, Peptides, and Recombinant Proteins**		

Cycloheximide	C7698	Sigma
3-Indolacetic acid (Auxin)	I2886	Sigma
Rapamycin	37094	Sigma
H-p-Bz-Phe-OH (BPA)	F2800.0005	Bachem
Cholesterol	700000	Avanti
1-palmitoyl-2-oleoyl-sn-glycero-3-phosphocholine (POPC)	850457	Avanti
1,2-dioleoyl-sn-glycero-3-phosphoethanolamine (DOPE)	850725	Avanti
1,2-dioleoyl-sn-glycero-3-phospho-L-serine (DOPS)	840035	Avanti
1,2-dioleoyl-sn-glycero-3-phosphoethanolamine-N-(lissamine rhodamine B sulfonyl) ammonium salt (18:1 Liss Rhod PE)	810150	Avanti
Decyl Maltose Neopentyl Glycol (DMNG)	NG322	Anatrace
Glyco-diosgenin (GDN)	GDN101	Anatrace
n-Dodecyl-β-D-Maltopyranoside (DDM)	D310	Anatrace
IGEPAL CA-630 (NP40)	I8896	Sigma
3x FLAG peptide	F4799	Sigma
His-ubiquitin	U530	Boston Biochem
DyLight 800 Maleimide	46621	Thermo Scientific
Peptide: Gly-Gly-Gly-Cys, counter ion -chloride	This study	Thermo Scientific
anti-HA magnetic beads	88837	Thermo Scientific
anti-FLAG M2 affinity gel	A2220	Sigma
anti-FLAG M2 magnetic beads	M8823	Sigma
Dynabeads Protein A	10002D	Thermo Fisher
High capacity streptavidin agarose resin	20361	Thermo Scientific
Streptavidin magnetic beads	88817	Thermo Scientific
NiNTA Agarose Beads	88222	Thermo Scientific
Detergent removal spin columns	87777	Thermo Scientific

**Deposited Data**		

Original gel images and western blots	This study; Mendeley Dataset	https://data.mendeley.com/datasets/37g4k2zvt9/draft?a=a91d9772-651b-4684-9d08-b7dab6849ecb

**Experimental Models: Organisms/Strains**		

*S. cerevisiae*: Strain background: BY4741	Research Genetics	S288C
*S. cerevisiae*: Strain background: BY4742	Research Genetics	S288C
*Mat a ura3-52 his3Δ200 leu2Δ1 trp1Δ63*	FY251	yPC1507
*Mat a ura3Δ0 his3Δ1 leu2Δ0 met15Δ0 asi1::KANR*	Foresti et al., 2014	yPC2008
*Mat a ura3Δ0 his3Δ1 leu2Δ0 met15Δ0 asi2::KANR*	Foresti et al., 2014	yPC2009
*Mat a ura3Δ0 his3Δ1 leu2Δ0 met15Δ0 asi3::KANR*	Foresti et al., 2014	yPC2010
*Mat a ura3Δ0 his3Δ1 leu2Δ0 met15Δ0 < pPC1230 >*	This study	yPC11635
*Mat a ura3Δ0 his3Δ1 leu2Δ0 met15Δ0 asi1::KANR < pPC1230 >*	This study	yPC11650
*Mat a ura3Δ0 his3Δ1 leu2Δ0 met15Δ0 asi2::KANR < pPC1230 >*	This study	yPC11636
*Mat a ura3Δ0 his3Δ1 leu2Δ0 met15Δ0 asi3::KANR < pPC1230 >*	This study	yPC11651
*Mat α his3Δ1 leu2Δ0 lys2Δ0 ura3Δ0 doa10::His5 < pPC1230 >*	This study	yPC11652
*Mat a ura3-52 his3Δ200 leu2Δ1 trp1Δ63 lys2Δ0 hrd1::HYGB < pPC1230 >*	This study	yPC11653
*Mat α his3Δ1 leu2Δ0 lys2Δ0 ura3Δ0 ubc7::HYGB < pPC1230 >*	This study	yPC11712
*Mat a his3Δ1 leu2Δ0 lys2Δ0 ura3Δ0 ubc4::KANR < pPC1230 >*	This study	yPC11713
*Mat ? his3Δ1 leu2Δ0 lys2Δ0 ura3Δ0 ubc7::HYGB ubc4::KANR < pPC1230 >*	This study	yPC11714
*Can1::Ste2pr-Leu2 Fpr1::Ura tor1-1 LYS+ Lyp1:: leu2Δ0 his3Δ1 ura3Δ0 Pil1-(6)-RFP-(24)-FKBP::NatR < pPC1356 >*	This study	yPC9650
*Can1::Ste2pr-Leu2 Fpr1::Ura tor1-1 LYS+ Lyp1:: leu2Δ0 his3Δ1 ura3Δ0 Esc1-RFP-(24)-FKBP::NatR < pPC1356 >*	This study	yPC9651
*Mat a ura3Δ0 his3Δ1 leu2Δ0 met15Δ0 < pPC1302 >*	This study	yPC11667
*Mat a ura3Δ0 his3Δ1 leu2Δ0 met15Δ0 asi1::KANR < pPC1302 >*	This study	yPC11668
*Mat a his3Δ1 leu2Δ0 met15Δ0 ura3Δ0 doa10::KANR < pPC1302 >*	This study	yPC11669
*Mat a ura3-52 his3Δ200 leu2Δ1 trp1Δ63 lys2Δ0 hrd1::HYGB < pPC1302 >*	This study	yPC11670
*Mat a ura3Δ0 his3Δ1 leu2Δ0 met15Δ0 < pPC1313 >*	This study	yPC11671
*Mat a ura3Δ0 his3Δ1 leu2Δ0 met15Δ0 asi1::KANR < pPC1313 >*	This study	yPC11672
*Mat a his3Δ1 leu2Δ0 met15Δ0 ura3Δ0 doa10::KANR < pPC1313 >*	This study	yPC11673
*Mat a ura3-52 his3Δ200 leu2Δ1 trp1Δ63 lys2Δ0 hrd1::HYGB < pPC1313 >*	This study	yPC11674
*Mat a ura3Δ0 his3Δ1 leu2Δ0 met15Δ0 < pPC1301 >*	This study	yPC11663
*Mat a ura3Δ0 his3Δ1 leu2Δ0 met15Δ0 asi1::KANR < pPC1301 >*	This study	yPC11664
*Mat a his3Δ1 leu2Δ0 met15Δ0 ura3Δ0 doa10::KANR < pPC1301 >*	This study	yPC11665
*Mat a ura3-52 his3Δ200 leu2Δ1 trp1Δ63 lys2Δ0 hrd1::HYGB < pPC1301 >*	This study	yPC11666
*Mat a ura3-52 his3Δ200 leu2Δ1 trp1Δ63 ubc7::HYGB NATR-ADHp-3xFlag-Asi1 < pPC1229+ pPC557 >*	This study	yPC10511
*Mat a ura3-52 his3Δ200 leu2Δ1 trp1Δ63 ubc7::HYGB NATR-ADHp-3xFlag-Asi1 < pPC1450+ pPC557 >*	This study	yPC10512
*Mat a ura3-52 his3Δ200 leu2Δ1 trp1Δ63 ubc7::HYGB NATR-ADHp-3xFlag-Asi1 < pPC1451+ pPC557 >*	This study	yPC10513
*Mat a ura3-52 his3Δ200 leu2Δ1 trp1Δ63 ubc7::HYGB NATR-ADHp-3xFlag-Asi1 < pPC1452+ pPC557 >*	This study	yPC10514
*Mat a ura3-52 his3Δ200 leu2Δ1 trp1Δ63 ubc7::HYGB NATR-ADHp-3xFlag-Asi1 < pPC1453+ pPC557 >*	This study	yPC10515
*Mat a ura3-52 his3Δ200 leu2Δ1 trp1Δ63 ubc7::HYGB NATR-ADHp-3xFlag-Asi1 < pPC1454+ pPC557 >*	This study	yPC10516
*Mat a ura3-52 his3Δ200 leu2Δ1 trp1Δ63 ubc7::HYGB NATR-ADHp-3xFlag-Asi1 < pPC1455+ pPC557 >*	This study	yPC10517
*Mat a ura3-52 his3Δ200 leu2Δ1 trp1Δ63 ubc7::HYGB NATR-ADHp-3xFlag-Asi1 < pPC1456+ pPC557 >*	This study	yPC10518
*Mat a ura3-52 his3Δ200 leu2Δ1 trp1Δ63 ubc7::HYGB NATR-ADHp-3xFlag-Asi1 < pPC1457+ pPC557 >*	This study	yPC10519
*Mat a ura3-52 his3Δ200 leu2Δ1 trp1Δ63 ubc7::KANR < pPC1229+ pPC557 >*	This study	yPC10502
*Mat a ura3-52 his3Δ200 leu2Δ1 trp1Δ63 ubc7::KANR < pPC1451+ pPC557 >*	This study	yPC10504
*Mat a ura3-52 his3Δ200 leu2Δ1 trp1Δ63 ubc7::KANR < pPC1453+ pPC557 >*	This study	yPC10506
*Mat a ura3-52 his3Δ200 leu2Δ1 trp1Δ63 ubc7::KANR < pPC1454+ pPC557 >*	This study	yPC10507
*Mat a ura3-52 his3Δ200 leu2Δ1 trp1Δ63 ubc7::KANR < pPC1456+ pPC557 >*	This study	yPC10509
*Mat a ura3-52 his3Δ200 leu2Δ1 trp1Δ63 ubc7::KANR asi1::HYGB < pPC1229+ pPC557 >*	This study	yPC10520
*Mat a ura3-52 his3Δ200 leu2Δ1 trp1Δ63 ubc7::KANR asi1::HYGB < pPC1451+ pPC557 >*	This study	yPC10521
*Mat a ura3-52 his3Δ200 leu2Δ1 trp1Δ63 ubc7::KANR asi1::HYGB < pPC1453+ pPC557 >*	This study	yPC10522
*Mat a ura3-52 his3Δ200 leu2Δ1 trp1Δ63 ubc7::KANR asi1::HYGB < pPC1454+ pPC557 >*	This study	yPC10523
*Mat a ura3-52 his3Δ200 leu2Δ1 trp1Δ63 ubc7::KANR asi1::HYGB < pPC1456+ pPC557 >*	This study	yPC10524
*Mat a ura3-52 his3Δ200 leu2Δ1 trp1Δ63 ubc7::KANR asi3::HYGB < pPC1229+ pPC557 >*	This study	yPC10525
*Mat a ura3-52 his3Δ200 leu2Δ1 trp1Δ63 ubc7::KANR asi3::HYGB < pPC1451+ pPC557 >*	This study	yPC10526
*Mat a ura3-52 his3Δ200 leu2Δ1 trp1Δ63 ubc7::KANR asi3::HYGB < pPC1453+ pPC557 >*	This study	yPC10527
*Mat a ura3-52 his3Δ200 leu2Δ1 trp1Δ63 ubc7::KANR asi3::HYGB < pPC1454+ pPC557 >*	This study	yPC10528
*Mat a ura3-52 his3Δ200 leu2Δ1 trp1Δ63 < pPC1229+ pPC557 >*	This study	yPC10493
*Mat a ura3-52 his3Δ200 leu2Δ1 trp1Δ63 < pPC1450+ pPC557 >*	This study	yPC10494
*Mat a ura3-52 his3Δ200 leu2Δ1 trp1Δ63 < pPC1451+ pPC557 >*	This study	yPC10495
*Mat a ura3-52 his3Δ200 leu2Δ1 trp1Δ63 < pPC1452+ pPC557 >*	This study	yPC10496
*Mat a ura3-52 his3Δ200 leu2Δ1 trp1Δ63 < pPC1453+ pPC557 >*	This study	yPC10497
*Mat a ura3-52 his3Δ200 leu2Δ1 trp1Δ63 < pPC1454+ pPC557 >*	This study	yPC10498
*Mat a ura3-52 his3Δ200 leu2Δ1 trp1Δ63 < pPC1455+ pPC557 >*	This study	yPC10499
*Mat a ura3-52 his3Δ200 leu2Δ1 trp1Δ63 < pPC1456+ pPC557 >*	This study	yPC10500
*Mat a ura3-52 his3Δ200 leu2Δ1 trp1Δ63 < pPC1457+ pPC557 >*	This study	yPC10501
*Mat ? ura3Δ0 his3Δ1 leu2Δ0 asi1::NATR asi2::HYGB asi3::KANR doa10::crispr hrd1::crispr < pPC1585 + pPC1583 + pPC1586 >*	This study	yPC11563
*Mat ? ura3Δ0 his3Δ1 leu2Δ0 asi1::NATR asi2::HYGB asi3::KANR doa10::crispr hrd1::crispr < pPC1417 + pPC1587*	This study	yPC11565
*Mat ? ura3Δ0 his3Δ1 leu2Δ0 asi1::NATR asi2::HYGB asi3::KANR doa10::crispr hrd1::crispr < pPC1417 + pPC1583 + pPC1587 >*	This study	yPC11566
*Mat ? ura3Δ0 his3Δ1 leu2Δ0 asi1::NATR asi2::HYGB asi3::KANR doa10::crispr hrd1::crispr < pPC1417 + pPC1583 + pPC1581 >*	This study	yPC11567
*Mat a ura3Δ0 his3Δ1 leu2Δ0 met15Δ0 < pPC1082 >*	Foresti et al., 2014	yPC8632
*Mat a ura3Δ0 his3Δ1 leu2Δ0 met15Δ0 asi2::KANR < pPC1082 >*	Foresti et al., 2014	yPC8634
*Mat a ura3-52 his3Δ200 leu2Δ1 trp1Δ63 Asi2(261-264Δ)::crispr < pPC1082 >*	This study	yPC11654
*Mat a ura3Δ0 his3Δ1 leu2Δ0 met15Δ0 NATR-ADHp-3xFlag-Asi2 < pPC1082 >*	This study	yPC11655
*Mat a ura3Δ0 his3Δ1 leu2Δ0 met15Δ0 NATR-ADHp-3xFlag-Asi2 (261-264Δ)::crispr < pPC1082 >*	This study	yPC11656
*Mat a ura3Δ0 his3Δ1 leu2Δ0 met15Δ0 wbp1-2::KANR*	Li et al., 2011	yPC8032
*Mat ? ura3Δ0 his3Δ1 leu2Δ0 met15Δ0 wbp1-2::KANR asi1::NATR*	This study	yPC8169
*Mat ? ura3Δ0 his3Δ1 leu2Δ0 met15Δ0 wbp1-2::KANR asi2::NATR*	This study	yPC8370
*Mat ? ura3Δ0 his3Δ1 leu2Δ0 met15Δ0 wbp1-2::KANR asi3::NATR*	This study	yPC8372
*Mat ? ura3Δ0 his3Δ1 leu2Δ0 met15Δ0 wbp1-2::KANR doa10::HIS*	This study	yPC8230
*Mat ? ura3Δ0 his3Δ1 leu2Δ0 met15Δ0 wbp1-2::KANR hrd1::HygB*	This study	yPC8233
*Mat ? ura3Δ0 his3Δ1 leu2Δ0 met15Δ0 wbp1-2::KANR ubc7::HYGB*	This study	yPC8227
*Mat a ura3Δ0 his3Δ1 leu2Δ0 met15Δ0 gpi8-ts::KANR*	Li et al., 2011	yPC8028
*Mat ? ura3Δ0 his3Δ1 leu2Δ0 met15Δ0 gpi8-ts::KANR asi1::NATR*	This study	yPC11719
*Mat ? ura3Δ0 his3Δ1 leu2Δ0 met15Δ0 gpi8-ts::KANR asi2::NATR*	This study	yPC8671
*Mat ? ura3Δ0 his3Δ1 leu2Δ0 met15Δ0 gpi8-ts::KANR asi3::NATR*	This study	yPC8677
*Mat ? ura3Δ0 his3Δ1 leu2Δ0 met15Δ0 gpi8-ts::KANR doa10::HIS*	This study	yPC8674
*Mat a ura3Δ0 his3Δ1 leu2Δ0 met15Δ0 gpi8-ts::KANR hrd1::HYGB*	This study	yPC8655
*Mat a ura3Δ0 his3Δ1 leu2Δ0 met15Δ0 gpi8-ts::KANR ubc7::HYGB*	This study	yPC8657
*Mat a ura3Δ0 his3Δ1 leu2Δ0 met15Δ0 wbp1-1::KANR*	Li et al., 2011	yPC8027
*Mat a ura3Δ0 his3Δ1 leu2Δ0 met15Δ0 wbp1-1::KANR asi1::NATR*	This study	yPC8605
*Mat a ura3Δ0 his3Δ1 leu2Δ0 met15Δ0 wbp1-1::KANR asi2::NATR*	This study	yPC8664
*Mat a ura3Δ0 his3Δ1 leu2Δ0 met15Δ0 wbp1-1::KANR asi3::NATR*	This study	yPC8666
*Mat a ura3Δ0 his3Δ1 leu2Δ0 met15Δ0 wbp1-1::KANR doa10::HIS*	This study	yPC8668
*Mat a ura3Δ0 his3Δ1 leu2Δ0 met15Δ0 wbp1-1::KANR hrd1::HYGB*	This study	yPC8652
*Mat a ura3Δ0 his3Δ1 leu2Δ0 met15Δ0 wbp1-1::KANR ubc7::HYGB*	This study	yPC8654
*Mat a ura3Δ0 his3Δ1 leu2Δ0 met15Δ0 lys2Δ0 can1Δ::LEU2-MFA1pr::His3 gpi16-ts::URA3*	Ben-Aroya et al., 2008	yPC9389
*Mat ? ura3Δ0 his3Δ1 leu2Δ0 met15Δ0 lys2Δ0 gpi16-ts::URA3 asi1::NATR*	This study	yPC9618
*Mat ? ura3Δ0 his3Δ1 leu2Δ0 met15Δ0 lys2Δ0 gpi16-ts::URA3 asi2::NATR*	This study	yPC9598
*Mat ? ura3Δ0 his3Δ1 leu2Δ0 met15Δ0 lys2Δ0 gpi16-ts::URA3 asi3::KANR*	This study	yPC9600
*Mat ? ura3Δ0 his3Δ1 leu2Δ0 met15Δ0 lys2Δ0 gpi16-ts::URA3 doa10::KANR*	This study	yPC9630
*Mat ? ura3Δ0 his3Δ1 leu2Δ0 met15Δ0 lys2Δ0 gpi16-ts::URA3 hrd1::KANR*	This study	yPC9632
*Mat ? ura3Δ0 his3Δ1 leu2Δ0 met15Δ0 lys2Δ0 gpi16-ts::URA3 ubc7::HYGB*	This study	yPC9622
*Mat a ura3-52 his3Δ200 leu2Δ1 trp1Δ63 URA3-GPDp-AtTIR1-9Myc Swp1-4AID-3xFlag-HYGB*	This study	yPC11735
*Mat a ura3-52 his3Δ200 leu2Δ1 trp1Δ63 URA3-GPDp-AtTIR1-9Myc Swp1-4AID-3xFlag-HYGB asi1::NATR*	This study	yPC11736
*Mat a ura3Δ0 his3Δ1 leu2Δ0 met15Δ0 lys2Δ0 can1Δ::LEU2-MFA1pr::His3 ost2-ts::URA3*	This study	yPC9391
*Mat ? ura3Δ0 his3Δ1 leu2Δ0 met15Δ0 lys2Δ0 ost2-ts::URA3 asi1::NATR*	This study	yPC11309
*Mat a ura3Δ0 his3Δ1 leu2Δ0 met15Δ0 stt3-7::KANR*	Li et al., 2011	yPC11259
*Mat ? ura3Δ0 his3Δ1 leu2Δ0 met15Δ0 stt3-7::KANR asi1::NATR*	This study	yPC11645
*Mat a ura3Δ0 his3Δ1 leu2Δ0 met15Δ0 lys2Δ0 can1Δ::LEU2-MFA1pr::His3 ost2-ts::URA3 OST4-3xFlag-HYGB*	This study	yPC11308
*Mat ? ura3Δ0 his3Δ1 leu2Δ0 met15Δ0 lys2Δ0 ost2-ts::URA3 asi1::NATR OST4-3xFlag-HYGB*	This study	yPC11310
*Mat a ura3Δ0 his3Δ1 leu2Δ0 met15Δ0 OST4-3xFlag-HYGB*	This study	yPC11661
*Mat a ura3Δ0 his3Δ1 leu2Δ0 met15Δ0 asi1::KANR OST4-3xFlag-HYGB*	This study	yPC11662
*Mat a ura3Δ0 his3Δ1 leu2Δ0 met15Δ0 < pPC1229 >*	This study	yPC11630
*Mat a ura3Δ0 his3Δ1 leu2Δ0 met15Δ0 asi1::KANR < pPC1229 >*	This study	yPC11646
*Mat a ura3Δ0 his3Δ1 leu2Δ0 met15Δ0 asi2::KANR < pPC1229 >*	This study	yPC11631
*Mat a ura3Δ0 his3Δ1 leu2Δ0 met15Δ0 asi3::KANR < pPC1229 >*	This study	yPC11647
*Mat α his3Δ1 leu2Δ0 lys2Δ0 ura3Δ0 doa10::His5 < pPC1229 >*	This study	yPC11648
*Mat a ura3-52 his3Δ200 leu2Δ1 trp1Δ63 lys2Δ0 hrd1::HYGB < pPC1229 >*	This study	yPC11649
*Mat α his3Δ1 leu2Δ0 lys2Δ0 ura3Δ0 ubc7::HYGB < pPC1229 >*	This study	yPC11715
*Mat a his3Δ1 leu2Δ0 lys2Δ0 ura3Δ0 ubc4::KANR < pPC1229 >*	This study	yPC11716
*Mat ? his3Δ1 leu2Δ0 lys2Δ0 ura3Δ0 ubc7::HYGB ubc4::KANR < pPC1229 >*	This study	yPC11717
*Mat a ura3Δ0 his3Δ1 leu2Δ0 met15Δ0 < pPC1356 >*	This study	yPC9635
*Mat a ura3Δ0 his3Δ1 leu2Δ0 met15Δ0 asi1::KANR < pPC1356 >*	This study	yPC9637
*Mat a ura3Δ0 his3Δ1 leu2Δ0 met15Δ0 SBP-TEV-ASI2:Crispr < pPC1229 >*	This study	yPC11756

**Oligonucleotides**		

Information on oligonucleotides is available upon request		

**Recombinant DNA**		

pRS316 - Erg11p-ERG11TM(Δ70-521)-3xHA	This study	pPC1230
pRS413-Erg11p-ERG11TM(Δ68-521)-sfGFP-FRB-HA	This study	pPC1356
pRS415-Voa1p-SP-3xHA-GPI8TM (376-411)	This study	pPC1302
pRS415-Voa1p-SP-3xHA-GPI16TM (542-610)	This study	pPC1313
pRS415-Voa1p-SP-3xHA-WBP1TM(387-430)	This study	pPC1301
pRS316 - Erg11p-TM(Δ58-521)-3xHA	This study	pPC 1229
TyrRS-tRNACUA	Chin et al., 2003	pESC-Bpa
pRS316 - Erg11p-ERG11TM F22 amber(TAG)(Δ58-521)-3xHA	This study	pPC1450
pRS316 - Erg11p-ERG11TM L27 amber(TAG)(Δ58-521)-3xHA	This study	pPC1451
pRS316 - Erg11p-ERG11TM I31 amber(TAG)(Δ58-521)-3xHA	This study	pPC1452
pRS316 - Erg11p-ERG11TM I36 amber(TAG)(Δ58-521)-3xHA	This study	pPC1453
pRS316 - Erg11p-ERG11TM F39 amber(TAG)(Δ58-521)-3xHA	This study	pPC1454
pRS316 - Erg11p-ERG11TM I43 amber(TAG)(Δ58-521)-3xHA	This study	pPC1455
pRS316 - Erg11p-ERG11TM L47 amber(TAG)(Δ58-521)-3xHA	This study	pPC1456
pRS316 - Erg11p-ERG11TM L51 amber(TAG)(Δ58-521)-3xHA	This study	pPC1457
pRS316 - Erg11p-ERG11-3xHA	Foresti et al., 2014	pPC1082
pRS423 - GAL1p-ASI1	This study	pPC1417
pRS425 -GAL1p-ASI3	This study	pPC1581
pRS423 - GAL1p-ASI1 (Δ562-624)	This study	pPC1585
pRS425 -GAL1p-ASI3 (Δ618-676)	This study	pPC1586
pRS426-GAL1p-SBP-TEV-ASI2	This study	pPC1583
pRS425 -GAL1p-3xFLAG-ASI3	This study	pPC1587
K27-T5p-HIS14-sumo-ERG11TM(Δ70-521)-MBP-LPTEGG	This study	pPC1822
K27-T5p-HIS14-sumo-ERG11TM(Δ70-521)(I36C)-MBP-LPTEGG	This study	pPC1823
K27-T5p-HIS14-sumo-MBP-UBC6TM (225-250)-HA-LPTEGG	This study	pPC1555
K27-T5p-HIS14-sumo-MPS3TM(149-182)-MBP-LPTEGG	This study	pPC1556
pET30-b-T7p-ASI1(424-616)-6xHIS	This study	pPC 1260
pET30-b-T7p-ASI2(153-271)-6xHIS	This study	pPC1234
pET30-b-T7p-ASI3(484-676)-6xHIS	This study	pPC 1262
K27-T5p-6xHIS-Sumo-UBC4		pPC 1878
pML107-GAPp-ASI2-gRNA1	This study	pPC1661
pML107-GAPp-HRD1-gRNA1	This study	pPC1695
pML107-GAPp-DOA10-gRNA1	This study	pPC1705

**Software and Algorithms**		

ImageJ	NIH	https://imagej.net/welcome
Image studio software Li-Cor	Li-Cor	https://www.licor.com/bio/image-studio-lite/
GraphPad Prism	N/A	https://www.graphpad.com/

### Lead Contact and Materials Availability

Further information and requests for reagents may be directed to and will be fulfilled by the Lead Contact, Pedro Carvalho (pedro.carvalho@path.ox.ac.uk). All unique/stable reagents generated in this study are available from the Lead Contact without restriction.

### Experimental Model and Subject Details

Yeast strains used in this study were *S. cerevisiae* derivatives of BY4741 or FY251. The genotypes of the strains and their mutant derivatives are listed in the Key Resources Table.

### Method Details

#### Yeast strains and Plasmids

The strains used are isogenic either to BY4741 (*Mata ura3Δ0 his3Δ1 leu2Δ0 met15Δ0*), BY4742 (*Matα his3Δ1 leu2Δ0 lys2Δ0 ura3Δ0*) or FY251 (*Mata ura3-52 his3Δ200 leu2Δ1 trp1Δ63*) and are listed in the Table S1. Tagging of proteins and individual gene deletions were performed by standard PCR-based homologous recombination (Longtine et al., 1998) or CRISPR (Laughery et al., 2015). The *Asi2Δ4* allele lacking residues (261-264) corresponding to amino acids Leucine, Cysteine, Leucine and Leucine, respectively, was generated by CRISPR-based gene editing as described (Laughery et al., 2015). In brief, a single guide RNA sequence targeting the desired region of Asi2 was designed using online software (http://wyrickbioinfo2.smb.wsu.edu) and cloned into a pML vector (Addgene) containing the Cas9 endonuclease from Streptococcus pyogenes using annealed oligos 3323, 3324. Cas9 plasmid along with a PCR amplified template containing the deletion of AA (261-264) were transformed using standard transformation protocol. Colonies were screened by PCR and sequencing of genomic DNA. Positive clones were grown in rich media (YPD) for 2-3 days to allow the loss of Cas9 plasmid. Guide RNAs are listed in the key resource table.

Strains with multiple gene deletions and temperature sensitive alleles were made by crossing haploid cells of opposite mating types, followed by sporulation and tetrad dissection using standard protocols (Guthrie and Fink, 1991).

Sanger sequencing was used to determine the sequence of the *wbp1-2* temperature sensitive allele. Analysis of several reactions consistently identified mutations leading to two amino acid changes- F249S and S297L- corresponding to residues in the ER luminal domain.

Plasmids used in this study are listed in the Key Resources Table and Table S1.

#### ERAD substrate degradation experiments

Cycloheximide (CHX) shutoff chases were essentially performed as described in Foresti et al. (2014). Briefly, yeast cells were grown either in rich media or synthetic media with 2% glucose. CHX was used at 250ug/ml from a stock of 12.5mg/ml. It was added to exponentially growing culture and 1 OD of cells was collected at the indicated time points. For anchor away technique, 10μM rapamycin was added 1 hour before the addition of CHX. Temperature sensitive mutants were grown at 25°C and shifted to the restrictive temperature as indicated in the corresponding figure legend. For auxin-dependent degradation, 0.8mM auxin was added 1 hour before the addition of CHX. 1 OD of cells were collected at the specified time points and whole cell lysates were prepared as described (Kushnirov, 2000) and analyzed by immunoblotting. Antibodies used in this study are listed in the key resource table.

Data quantification was performed using Image Studio software (Li-Cor) or ImageJ and graphs were plotted in Prism. Representative images of three independent experiment are shown.

#### *In vivo* site-specific crosslinking

Cells with various genotypes were transformed with two plasmids. One encoding both for a modified tRNA synthetase capable of charging the unnatural amino acid benzoyl phenylalanine (BPA) on a tRNA as well as amber stop codon suppressor tRNA. The second plasmid encoded for TM56-HA with individual amber codons. Cells carrying both plasmids were grown in synthetic minimal media and diluted overnight into 100ml of the same media. At 0.3-0.4 OD_600_, BPA was added to a final concentration of 0.3mM (from a 0.2M in 1M NaOH freshly prepared stock). For BPA incorporation, cells were grown for additional 5-6 hours at 25°C. Cells were harvested by a centrifuge spin for 2 min at 3000 g and resuspended in 1ml of cold water. Half of cells were transferred to a 12 well plate and subjected to UV irradiation for 1 hour at 4°C using a B-100AP lamp (UVP, CA). The other half of the cells was incubated on ice and served as non-irradiated control. After UV irradiation, cells were harvested by centrifuge spin for 2 min at 3000 g. Both irradiated and control cells were lysed in LB buffer (50mM Tris/HCl [pH7.4], 200mM NaCl, 1mM EDTA, 2mM phenylmethylsulfonyl fluoride(PMSF) and protease inhibitor cocktail) by 5-6 × 1 min cycles of bead beating. Lysates were cleared by a 10 min centrifugation at 600 g. Cleared lysates were centrifuged at 100000 g (25 min at 4°C) to obtain crude membrane fractions. The membrane pellet was resuspended in denaturing buffer (50mM Tris/HCl [pH7.4], 1mM EDTA, 1% SDS, 2M urea) and solubilized at 65°C for 30-40 min. Unsolubilized material was pelleted by centrifugation (15 min spin at 13000 g). The solubilized material was diluted with LB supplemented with 1% Nonidet P-40 and incubated overnight with anti-HA beads (Pierce TM). Beads were washed 3 times with LB/1% Nonidet P-40 and bound proteins eluted with SDS buffer and analyzed by immunoblotting.

#### Native Immunoprecipitation

Approximately 100OD of cells grown in YPD were harvested by centrifugation at 3000 g and washed with LB buffer (50mM Tris/HCl [pH7.4], 200mM NaCl, 1mM EDTA, 2mM phenylmethylsulfonyl fluoride(PMSF) and protease inhibitor cocktail). Lysates and crude membrane fractions were prepared as described above. Detergent extracts were prepared by solubilizing crude membrane fractions in LB/1% glyco-diosgenin (GDN) (Generon) or 1% decyl maltose neopentyl glycol (DMNG). Unsolubilized material was cleared by a 15 min spin at 13000 g. The cleared detergent extracts were incubated overnight at 4°C with FLAG M2 magnetic beads (Sigma-Aldrich) or Protein A beads (Thermo) coupled to anti-Asi1 and Anti-Asi2 antibodies. Beads were washed 3 times with LB/1% GDN or 1% DMNG, eluted with SDS buffer and analyzed by immunoblotting. The input corresponds to 10% of the total extract used for IP.

#### Growth assays

Cells with the relevant genotypes growing on YPD plates were inoculated in 5 mL of YPD and grown overnight at 25C to an OD600∼3. Six 10-fold serial dilutions were performed in YPD. 3 μl of the dilutions were spotted on YPD agar plates and incubated at the respective restrictive temperature for 2-3 days.

#### Expression and purification of the Asi complex from *S. cerevisiae*

All three Asi proteins were co-expressed and purified essentially as described (Stein et al., 2014). Briefly, cells lacking *ASI1*, *ASI2*, *ASI3*, *HRD1* and *DOA10* were transformed with high copy plasmids from the pRS42X series (Mumberg et al., 1994) encoding untagged Asi1 and Asi3 as well as Asi2 fused to a N-terminal SBP (Streptavidin binding peptide) (Keefe et al., 2001) followed by a TEV protease cleavage site. A codon optimized version of ASI3 was used. In some cases, an N-terminal FLAG-tagged Asi3 version was used. Cells were inoculated in Synthetic Drop-out media with 2% (w/v) glucose and grown for 24 hr at 30°C. Cells were diluted 1:40 in fresh medium and incubated for additional 24 hr. Protein expression was induced by addition of 8% (w/v) galactose in 4x YEP broth and incubated for 12 hr. Cell pellets (∼90 g) were harvested by centrifugation and washed with water and buffer A (20mM HEPES, 2mM magnesium acetate, 150mM potassium chloride). Cells were resuspended in 140ml of buffer A with 1mM PMSF and 1.5μM of pepstatin A and transferred to a bead beater chamber (BioSpec) containing ∼150 g glass beads (0.5mM diameter from BioSpec). Bead beater chamber was assembled with an ice water jacket. Lysis was induced by 40 cycles of 30’’ on/off. Glass beads were removed by filtration and lysates cleared by low-speed spinning at 2000xg for 18 min. The supernatant was transferred to Ti45 tubes and crude membranes were prepared by centrifugation (40,000 rpm for 45 min). Membranes were washed twice with buffer B (20mM HEPES, 2mM Magnesium acetate, 300mM sodium chloride, 200mM sucrose). The membrane pellet was solubilized for 90 min in 180ml of buffer B supplemented with 1%(w/v) of DMNG, 1mM PMSF, 1.5μM Pepstatin A, 200ul 14,3M of β-mercaptoethanol. Non-solubilized material was removed by centrifugation in Ti45 tubes for 30 min at 40000 rpm. 3 mL of High Capacity Streptavidin Agarose resin (Pierce) or FLAG M2 agarose beads (Sigma) was added to detergent solubilized extract and incubated overnight. After incubation, the material was transferred to 20 mL gravity columns and beads were washed with 25 column volumes of buffer C (20mM HEPES, 2mM Magnesium acetate, 300mM sodium chloride, 0.3mM DMNG) by gravity flow. Bound proteins were eluted with buffer C containing 2mM Biotin (or with 0.15 μg/ml 3xFlag-peptide). Eluted material was concentrated using 50kDa cut off centrifugal filters (Amicon Ultra, Merck) and snap frozen in liquid nitrogen until use.

#### Preparation of Liposomes

1-palmitoyl-2-oleoyl-*sn*-glycero-3-phosphocholine (POPC), 1,2-dioleoyl-*sn*-glycero-3-phosphoethanolamine (DOPE), 1,2-dioleoyl-*sn-*glycero-3-phospho-L-serine (DOPS), cholesterol and 1,2-dioleoyl-sn-glycero-3-phosphoethanolamine-N-(lissamine rhodamine B sulfonyl) ammonium salt (18:1 Liss Rhod PE) were obtained from Avanti lipids and dissolved in chloroform. Lipids were mixed at a percentage of (64.5:20:10:5:0.5). The chloroform was evaporated in a Rotavap until a lipid film was obtained. Lipids were resuspended in 1ml of diethylether and 300ul of an aqueous buffer containing 20mM HEPES/KOH (pH 7.4), 50mM potassium chloride, 5mM magnesium acetate, sonicated for 1 min and the volume adjusted to 2ml with the aqueous buffer. Diethylether was evaporated for 3-4 hr using a Rotavap. Liposomes were extruded through 400nm size filters (11x) and 100 nm filters (21x).

#### Reconstitution into proteoliposomes

Freshly prepared liposomes were partially solubilized using 2mM of DMNG for 30 min on ice. Subsequently 2.3μM of the purified ASI complex, 0.5μM of TM68 or 0.5 μM Mps3TM or 0.5 μM Ubc6TM were added and incubated for 1hr on ice. The mixture was applied to detergent removal spin columns (Pierce). This step was repeated 2 times and the final proteoliposomes were used for the *in vitro* assays.

#### Ubiquitination Assay

Proteoliposomes were added to the ubiquitination reaction mix containing buffer U (20mM HEPES (pH 7.4), 2mM magnesium acetate, 150mM sodium chloride, 0.5mM TCEP), 0.2mg/ml BSA 0.23 μM Uba1p, 2.1 μM Ubc4p, 2.3 μM Ubc7p, 2.2 μM Cue1p, 122.5 μM His-ubiquitin (Boston Biochem, from yeast), 2.5mM ATP. Reactions were incubated at 30°C for 1 hr. Samples were either analyzed directly by SDS-PAGE and fluorescence scanning using Li-Cor image or subjected to His-Ub pull down to enrich for ubiquitin conjugates. In the latter, reactions were solubilized in buffer D (20mM HEPES (pH 7.4), 2mM magnesium acetate, 150mM sodium chloride, 6M urea, 10mM imidazole, 1% Triton X-100) for 15 min at 4°C followed by incubation with Ni-NTA agarose beads (Pierce) for 3 hr at 4°C. Beads were washed five times with buffer E (20mM HEPES (pH 7.4), 2mM magnesium acetate, 150mM sodium chloride, 6M urea, 20mM imidazole, 1% Triton X-100). Bound ubiquitin conjugates were eluted using SDS buffer supplemented with 500mM Imidazole at 65°C for 10 min. Samples were analyzed by SDS-PAGE and fluorescence scanning using Li-Cor imager.

#### Membrane extraction assay

Freshly prepared proteoliposomes containing TM68 and Asi complex were immobilized via SBP-Asi2 using streptavidin magnetic beads (Pierce) in buffer U (20mM HEPES (pH 7.4), 2mM magnesium acetate, 150mM sodium chloride, 0.5mM TCEP). Bound proteoliposomes were ubiquitinated as described for the ubiquitination assay. The ubiquitination mix was removed and the beads containing ubiquitinated species were incubated for 30 min at 30°C with buffer U supplemented with 500mM Cdc48, 500mM Ufd1/Npl4 and 2.5mM ATP. The supernatant containing the extracted proteins was collected and subjected to a His-Ub pulldown, elution, and analysis were performed as described above for the ubiquitination assay.

#### Nycodenz floatation assay

Proteoliposomes (25μl) were mixed with 25μl of 80% of nycodenz solution in buffer U and overlayed with 50μl of 30% nycodenz, 50μl of 15% nycodenz and 50μl of buffer U. It was subjected to centrifugation in TLS55 rotor at 55000 rpm for 1 hr at 4°C. Fractions were collected from the top layer, mixed with SDS buffer and analyzed by SDS-PAGE followed by immunoblotting and fluorescence scanning.

#### *In vitro* crosslinking

Reconstituted proteoliposomes were incubated with 6mM of 1,4-bismaleimidobutane (BMB) for 1 hr at RT. Reactions were stopped by the addition of SDS buffer and analyzed by SDS-PAGE and immunoblotting.

#### Recombinant protein expression and purification

TM68-MBP was expressed and purified from E. Coli. Plasmids containing N-terminal His14-Sumo-tag followed by TM68, the maltose binding peptide (MBP) and a sortase recognition sequence (LPTEGG) were transformed into BL21-CodonPlus (DE3)-RIPL (Agilent Technologies) cells. Cells were grown overnight in LB media containing Kanamycin. Next morning, cells were diluted into terrific broth with Kanamycin and protein expression was induced by the addition of 0.5mM of IPTG and grown for additional 3 hr at 30°C. Cells were harvested, washed with water and resuspended in buffer 20mM Tris-HCl (pH 8), 500mM NaCl, 20mM imidazole, pH 8.0, 1mM PMSF. Cells were lysed by passing through the microfluidizer (5x, 10000 psi). The lysate was cleared by centrifugation at 4000 rpm for 10 min. Cleared lysates were subjected to ultracentrifugation in Ti45 tubes for 45 min at 40,000 rpm to isolate a crude membrane fraction. Membranes were resuspended in buffer W (50mM Tris-HCl (pH 8.0), 500mM NaCl, 30mM imidazole,1mM PMSF) supplemented with 1% DDM and solubilized for 90 min. Non solubilized material was removed by centrifugation in Ti45 tubes for 30 min at 40000 rpm. 3 mL of Ni-NTA Agarose beads (Thermo) was added to detergent solubilized extract and incubated for 3hr at 4°C. After incubation, the material was transferred to a 20ml gravity column and beads were washed by gravity flow with 20 column volumes of buffer W with 1% DDM and 20 column volumes of cleavage buffer (20mM Tris-HCl (pH 8.0), 200mM NaCl, 10mM imidazole,) supplemented with 1% DDM. Washed agarose beads were resuspended in 10ml of the cleavage buffer, transferred to a tube containing 1μM of sumo protease Ulp1 (Frey and Görlich, 2014) and incubated overnight at 4°C. Beads were pelleted by centrifugation at 2000 rpm for 3 min and TM68-MBP was collected in the supernatant. This step was repeated twice with fresh cleavage buffer. Supernatant from all the three centrifuge spins were pooled, concentrated using 30kDa cut off centrifugal filters (Amicon Ultra, Merck). Concentrated protein was loaded on to a Superdex 200 column (GE) equilibrated with buffer (20mM HEPES (pH 7.4), 200mM NaCl, 0.3mM DMNG). Peak fractions were collected and concentrated again. TM68 was labeled with Dy800 dye using sortase as described in Stein et al. (2014). Labeled TM68 was repurified on a Superdex 200 column (GE) equilibrated with buffer (20mM HEPES (pH 7.4), 200mM NaCl, 0.3mM DMNG), peak fractions were collected and snap frozen until use.

TM68(I36C) was purified as above except that 0.4mM TCEP was added to all the buffers and it was not labeled.

For Mps3TM purification, plasmid containing N-terminal His14-Sumo-tag followed by Mps3TM (residues 149-182), the maltose binding peptide (MBP) and a sortase recognition sequence (LPTEGG) were transformed into BL21-CodonPlus (DE3)-RIPL (Agilent Technologies) cells. Purification procedure was same as TM68-MBP.

For Ubc6TM purification, plasmid containing N-terminal His14-Sumo-tag followed by the maltose binding peptide (MBP)- Ubc6TM (residues 225-250) and a sortase recognition sequence (LPTEGG) were transformed into BL21-CodonPlus (DE3)-RIPL (Agilent Technologies) cells. It was purified as TM68 except that after cell lysis and ultracentrifugation step to isolate a crude membrane fraction, soluble supernatant fraction was used to purify Ubc6TM with the same buffers without detergent.

Uba1, Ubc7, Cue1, Cdc48, Npl4-Ufd1 were purified as described (Stein et al., 2014). For Ubc4 purification, a plasmid containing N-terminal His14-Sumo-tag followed by coding sequence of Ubc4 was transformed into BL21-CodonPlus (DE3)-RIPL (Agilent Technologies) cells. Cells were grown and protein expression induced by IPTG as described above. Cells were harvested, washed with water and resuspended in buffer 20mM Tris-HCl (pH 8), 500mM NaCl, 30mM imidazole, pH 8.0, 1mM PMSF. Cells were lysed by passing through the microfluidizer (2x, 18000 psi). Lysate was cleared by a centrifugation spin at 4000 rpm for 10 min. Cleared lysates were subjected to ultracentrifugation in Ti45 tube for 30 min at 40,000 rpm. 3ml of Ni-NTA Agarose beads (Thermo) was added to the soluble fraction and incubated for 3 hr at 4°C. After incubation, the material was transferred to a 50ml gravity column and beads were washed with 4 column volumes of buffer W and 4 column volumes of cleavage buffer supplemented with 200mM sucrose by gravity flow. Sumo protease treatment was carried on as described above for TM68-MBP. Concentrated protein was subjected to dialysis with buffer (20mM Tris-HCl (pH 8), 100mM NaCl, 1mM EDTA, 1mM DTT, 200mM sucrose) to remove imidazole and snap frozen until use.

### Quantification and Statistical Analysis

Data quantification was performed using Image Studio software (Li-Cor) or ImageJ and graphs were plotted in Prism. Representative images of at least three independent experiments are shown.

### Data and Code Availability

Raw data generated in this study and used in the preparation of the figures has been deposited as Mendeley Dataset and is available at https://data.mendeley.com/datasets/37g4k2zvt9/draft?a=a91d9772-651b-4684-9d08-b7dab6849ecb.

## Supporting Citations

The following references appear in the Supplemental Information: Hessa et al., 2005, Hessa et al., 2007, Tsirigos et al., 2015, Zhao and London, 2006.

## Supplemental Information

Document S1. Figures S1–S6 and Table S1

Document S2. Article plus Supplemental Information
