# Supplementary material for: Quality Control of Protein Complex Assembly by a Transmembrane Recognition Factor
Source: Mol Cell. 2020 Jan 2;77(1):108–119.e9. doi: 10.1016/j.molcel.2019.10.003 (PMC6941229; doi:10.1016/j.molcel.2019.10.003)
Supplement: Document S1. Figures S1–S6 and Table S1 [file mmc1.pdf]

Molecular Cell, Volume *77*

Supplemental Information

Quality Control of Protein Complex Assembly by a Transmembrane Recognition Factor

Nivedita Natarajan, Ombretta Foresti, Kim Wendrich, Alexander Stein, and Pedro Carvalho

Supplemental Information

Includes:

- Legends to supplemental figures
- Supplemental figures (S1 to S6)
- Supplemental Table S1

Legends to the supplemental figures

Figure S1. Degradation of unassembled subunits of ER complexes requires the Asi complex, related to Figure 1.

(A) Schematic representation of the glycosylphosphatidylinositol transamidase (GPI-T) complex essential for GPI anchor attachment to proteins. Individual subunits are indicated with different colors.

(B) The turnover of the GPI-T subunit Gpi8 was followed after inhibition of protein synthesis by CHX in WT and *asi1Δ* cells. Whole-cell extracts were analyzed by SDS–PAGE and western blotting. Arrowheads indicate the double band of Gpi8, detected with α-Gpi8 antibody and α-Pgk1 was used as loading control.

(C) The degradation of mutant Gpi8-ts, encoded by a temperature sensitive allele of *GPI8*, was followed after inhibition of protein synthesis with CHX in cells with the indicated genotypes after a 60 min shift to 37˚C. Whole cell extracts were analyzed by SDS-PAGE and western blotting. Gpi8 was detected with α-Gpi8 antibody and Dpm1 was used as loading control. The graph (right) shows the quantification of at least three independent experiments.

(D) Serial dilutions of cells with the indicated genotype were spotted on YPD and incubated for 2 days at 25˚C and 37˚C.

(E) The integrity of OST complex is disrupted in cells with ost2-ts allele. Complex integrity was analyzed by testing interaction between Wbp1 and Ost4-Flag by immunoprecipitation. Cells with the indicated genotypes were shifted to 37˚C for 90min prior lysis. Ost4-FLAG was immunoprecipitated with FLAG beads from a crude membrane fraction solubilized with 1% GDN. Eluted proteins were analyzed by SDS–PAGE and immunoblotting.

Wbp1 and Ost4 were detected with α-Wbp1 and α-Flag antibodies, respectively.

Figure S2. Erg11 transmembrane domain is degraded in a Asi-dependent manner, related to Figure 2.

(A) Amino acid sequence of the Erg11 truncations TM56 and TM68 indicating the various modules of the construct: the ER luminal amphipathic helix (residues 6-23), transmembrane region (residues 27-51) followed by a linker and a 3xHA tag.

(B) TM56 and TM68 are membrane associated. Crude membrane (M) and soluble (S) fractions from WT cells expressing TM56 and TM68 were prepared and analyzed by SDS-PAGE and immunoblotting. The membrane Dpm1 and the soluble Pgk1 proteins were used as controls.

(C) TM56-HA is a substrate of the Asi complex. TM56-HA degradation was analyzed as in Figure 2B.

(D) The degradation TM56-HA was analyzed as in (B) in *WT* cells or in cells with the indicated deletions.

(E) The degradation TM68-sfGFP-FRB-HA was analyzed as in (B) in *WT* and *asi1Δ* cells.

Figure S3. Degradation and features of transmembrane segments degraded by Asi-mediated ERAD, related to Figure 3.

(A) The degradation of HA-Gpi8 TM was followed after inhibition of protein synthesis by CHX in *WT* cells or in cells with the indicated deletions. Whole-

cell extracts were analyzed by SDS–PAGE and western blotting. It was detected with α-HA antibodies. Pgk1 was used as loading control and detected with α-Pgk1 antibody.

(B) The degradation of HA-Gpi16 TM was analyzed as in (A).

(C) The degradation of HA-Wbp1 TM was analyzed as in (A).

(D) The sequence of Erg11 TMD was determined by (Monk et al., 2014). Topcons (http://topcons.cbr.su.se/pred/) was used to predict the sequence of additional TMDs (Tsirigos et al., 2015). The sequence of the other TMDs and free energy of transmembrane insertion in the ER membrane (ΔG) was calculated according to http://dgpred.cbr.su.se/index.php?p=home (Hessa et al., 2005, 2007). Transmembrane hydrophobicity was calculated according to (Zhao and London, 2006). Net Charge was calculated based on the charge of individual amino acid at pH7.

Figure S4. TM56 crosslinks to Asi2, related to Figure 4.

(A) Steady state levels of endogenous Asi2 and Asi3 in cells with the indicated genotypes. Whole-cell extracts were analyzed by SDS–PAGE and immunoblotting using α-Asi2 and α-Asi3 antibodies. Dpm1 was used as a loading control and detected with α-Dpm1 antibodies.

(B) *WT* cells expressing plasmid-borne TM56 with the photoreactive amino acid analog benzoyl phenylalanine (BPA) at the indicated positions were subjected to UV irradiation. Non-irradiated cells were used as controls. Detergent-solubilized membranes were subjected to immunoprecipitation

with HA antibodies, and bound proteins were analyzed by SDS-PAGE and immunoblotting with HA and Asi2 antibodies.

Figure S5. Characterization of the purified ERAD components and Substrates, related to Figure 5.

(A) Asi complex and derivatives were overexpressed in *S. cerevisiae*, affinity purified and subjected to SDS-PAGE followed by staining with Coomassie blue (top) or immunoblotting. Asi3 and Asi2 were detected with α-Asi3 and α-SBP antibodies, respectively.

(B) The fusion protein SBP-TEV-Asi2 promotes the degradation of TM56. The degradation TM56-HA was followed after inhibition of protein synthesis by CHX in cells with the indicated genotype. Whole-cell extracts were analyzed by SDS–PAGE and immunoblotting. TM56 and Dpm1 were detected with α-HA and α-Dpm1 antibodies, respectively.

(C) The indicated ERAD components and the TM68-MBP substrate were recombinantly expressed, purified and subjected to SDS-PAGE, followed by Coomassie blue staining.

(D) Comparison of TM68-MBP ubiquitination in detergent and proteoliposomes. Ubiquitination reactions were performed as described in Figure 5B either in proteoliposomes (PLs) with Asi complex and TM68-MBP or in detergent (DMNG). Reactions were either directly analyzed by SDS PAGE and fluorescence scanning (Top) or subjected to His-ubiquitin affinity purification to enrich for ubiquitin conjugates. Line scan fluorescence intensity profiles are graphed on the right.

(E) Asi complexes and TM68-MBP were efficiently reconstituted into proteoliposomes. Asi complexes purified either with SBP-Asi2 or FLAG-Asi3 and TM68-MBP were subjected to nycodenz gradient (0-40%) centrifugation and analyzed by SDS-PAGE followed by fluorescence scanning (TM68) and immunoblotting for Asi3 and Asi2 with α-Asi3 and α-SBP antibodies, respectively

(F) Assessing the topology of Asi complex reconstituted in proteoliposomes. Proteoliposomes containing Asi complex in which Asi2 contains a TEV cleavage site in its N-terminal cytoplasmic domain (SBP-TEV-Asi2) were subjected to TEV protease (0.5ug) digestion in presence or absence of detergent (2mM DMNG) for 60 min at RT. Reactions were analyzed by SDS-PAGE and immunoblotting for SBP-TEV-Asi2 with α-SBP antibody.

(G) Proteoliposomes containing Asi complex and MBP fusions to the the indicated transmembrane segments (TM68, Mps3TM and Ubc6TM) were incubated with Ubi Mix for 60 minutes in presence or absence of ATP. Reactions were either analyzed directly by SDS-PAGE and fluorescence scanning (TM) (bottom panel), or subjected to His-ubiquitin affinity purification. Eluted proteins were analyzed by SDS-PAGE and fluorescence scanning (Top panel; ^{Ub}TM). Line scan fluorescence intensity profiles are graphed on the right. Asterisks (*) indicate dimers of Mps3TM-MBP.

(H) Asi complex and the individual substrates analyzed were efficiently reconstituted into proteoliposomes as assayed by floatation experiment. Purified Asi complex reconstituted with TM68-MBP, Mps3TM-MBP and

MBP-Ubc6TM were subjected to nycodenz gradient (0-40%) centrifugation and analyzed by SDS-PAGE followed by fluorescence scanning (TM) and immunoblotting for Asi3 and Asi2 with α-FLAG and α-SBP antibodies, respectively.

Figure S6. Characterization of Asi2 as a transmembrane domain recognition factor, related to Figure 6.

(A) Co-reconstitution of Asi complex and TM68-MBP only occurs in P1 proteoliposomes (as indicated in schematic representation above). Proteoliposomes were immobilized in streptavidin beads at RT for 90 min. Beads were washed and bound material was eluted with SDS buffer supplemented with biotin (E). Input and unbound are indicated as In and U, respectively. Samples were analyzed by SDS PAGE followed by immunoblotting for Asi2 with α-SBP antibody and fluorescence scanning for TM68.

(B) TM68 (I36C)-MBP is efficiently ubiquitinated by the Asi complex *in vitro*. Proteoliposomes containing Asi complex with TM68 (I36C) were used in ubiquitination reaction and analyzed as described in Figure 5C.

(C) The mutant protein Asi2Δ4 assembles with Asi1 and Asi3 into an intact Asi complex. Crude membrane fractions from cells with the indicated genotypes were solubilized with 1% DMNG and detergent extracts subjected to immunoprecipitation with α-Asi1 and α-Asi2 antibodies. Eluted proteins were analyzed by SDS–PAGE and immunoblotting. Asi1, Asi2 and Asi3 were detected with α-Asi1, α-Asi2 and α-Asi3 antibodies, respectively. Asterisks (*) denote non-specific bands. Note that Asi1

protein is only detected in the IP lanes as in the lysates Asi1 signal is obscured by two strong non-specific bands.

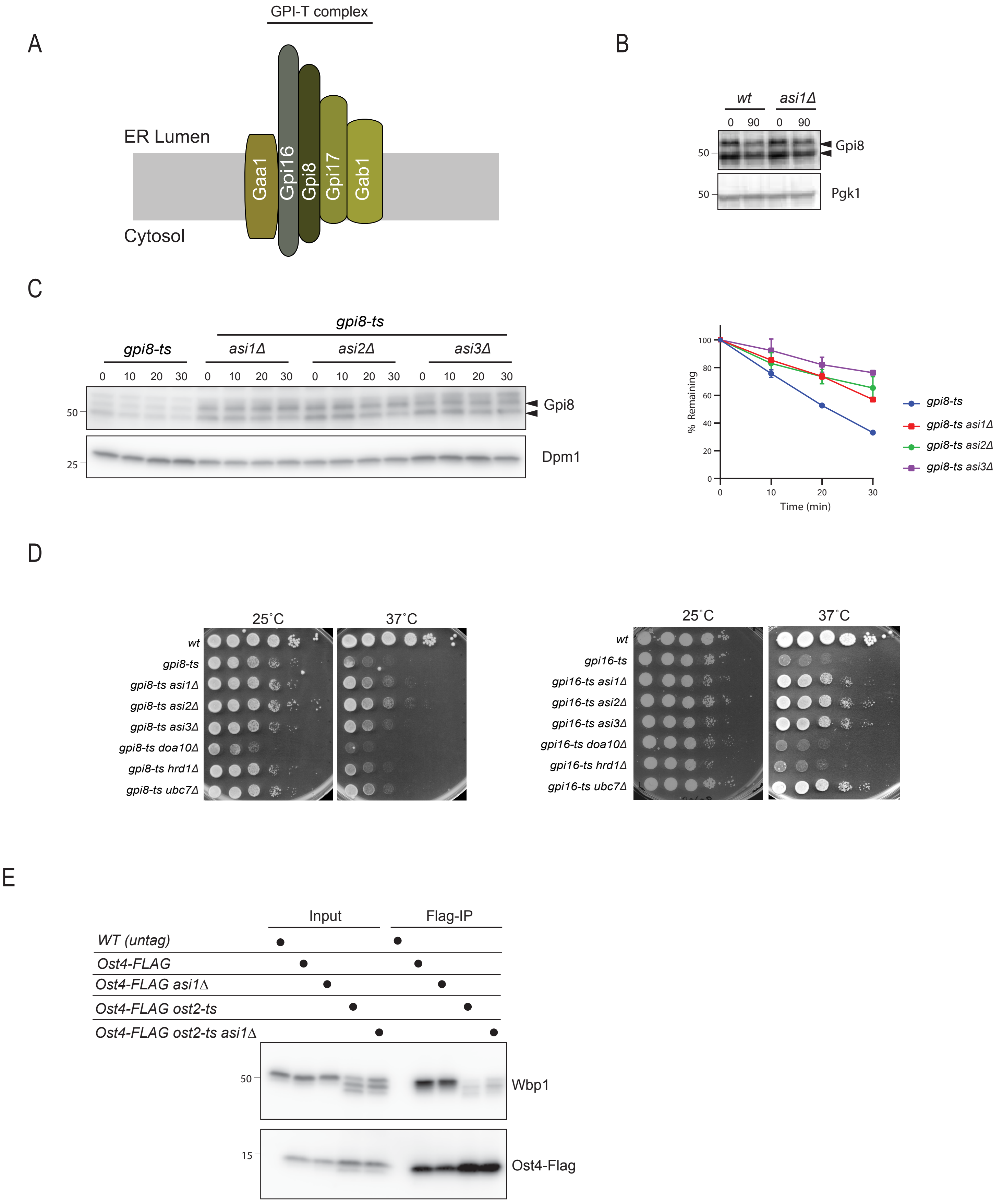
A
GPI-T complex
ER Lumen
Cytosol
Gaa1
Gpi16
Gpi8
Gpi17
Gab1
B
wt
asi1Δ
0 90 0 90
50
Gpi8
50
Pgk1
C
gpi8-ts
gpi8-ts
asi1Δ
asi2Δ
asi3Δ
0 10 20 30 0 10 20 30 0 10 20 30 0 10 20 30
50
Gpi8
25
Dpm1
% Remaining
Time (min)
gpi8-ts
gpi8-ts asi1Δ
gpi8-ts asi2Δ
gpi8-ts asi3Δ
D
25˚C
37˚C
wt
gpi8-ts
gpi8-ts asi1Δ
gpi8-ts asi2Δ
gpi8-ts asi3Δ
gpi8-ts doa10Δ
gpi8-ts hrd1Δ
gpi8-ts ubc7Δ
25˚C
37˚C
wt
gpi16-ts
gpi16-ts asi1Δ
gpi16-ts asi2Δ
gpi16-ts asi3Δ
gpi16-ts doa10Δ
gpi16-ts hrd1Δ
gpi16-ts ubc7Δ
E
Input
Flag-IP
WT (untag)
Ost4-FLAG
Ost4-FLAG asi1Δ
Ost4-FLAG ost2-ts
Ost4-FLAG ost2-ts asi1Δ
50
Wbp1
15
Ost4-Flag

Figure S2 Natarajan et al.

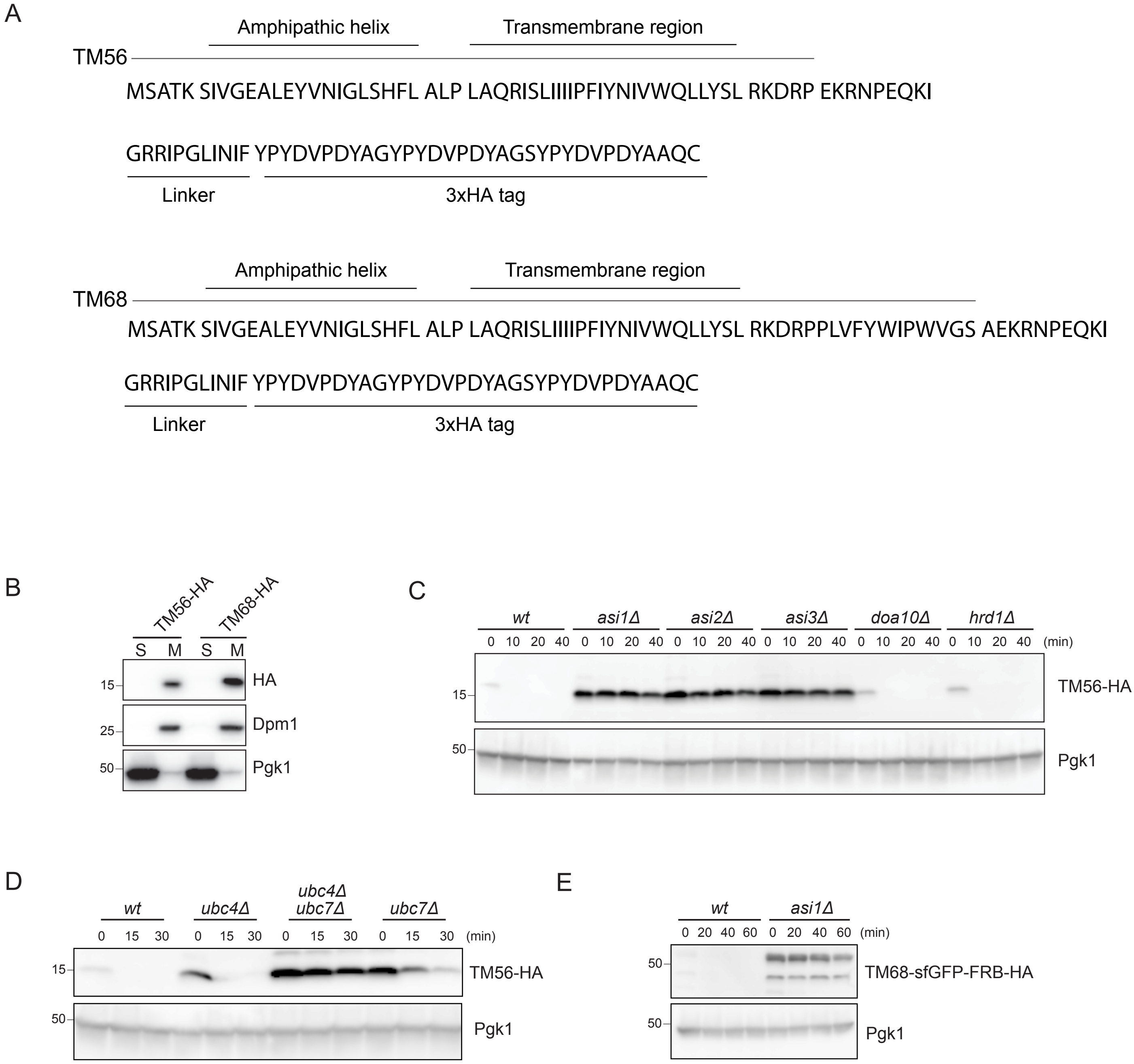

Figure S3 Natarajan et al.

A

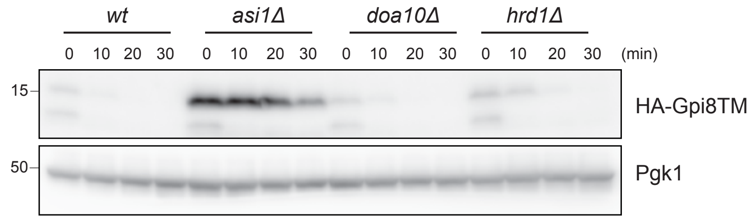

B

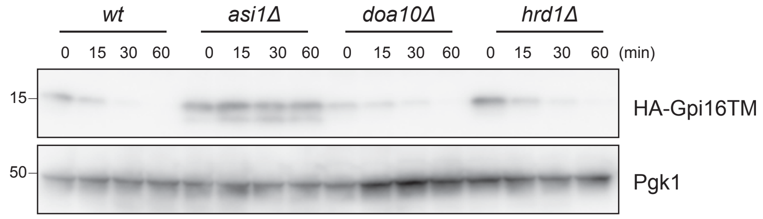

C

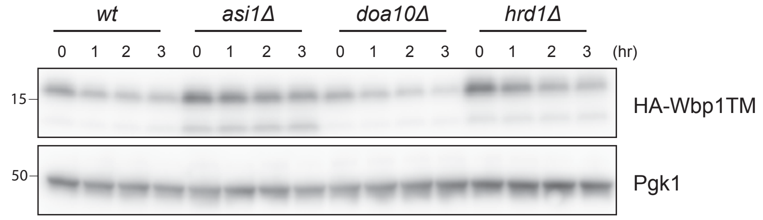

D

TM	Sequence	Length	ΔG (kcal/mol)	Hydrophobicity	Net charge at pH7
Erg11	LAQRISLIIIIPFIYNIVWQLLYSL	25	- 0.281	18.85	1
Gpi8	FKQSATIILALIVTILWFML	20	-1.393	17.8	1
Gpi16	YNVIILTSTIMGLIFGMLYNL	21	0.419	17.59	0
Wbp1	WVYISAICGVIVAWIFFVVSFVT	23	-1.76	25	-0.1

A

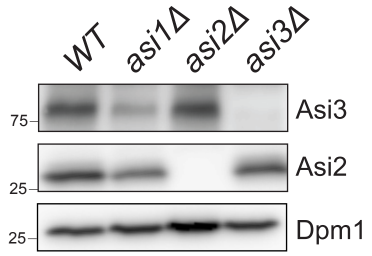
WT
asi1Δ
asi2Δ
asi3Δ
75
Asi3
25
Asi2
25
Dpm1

B

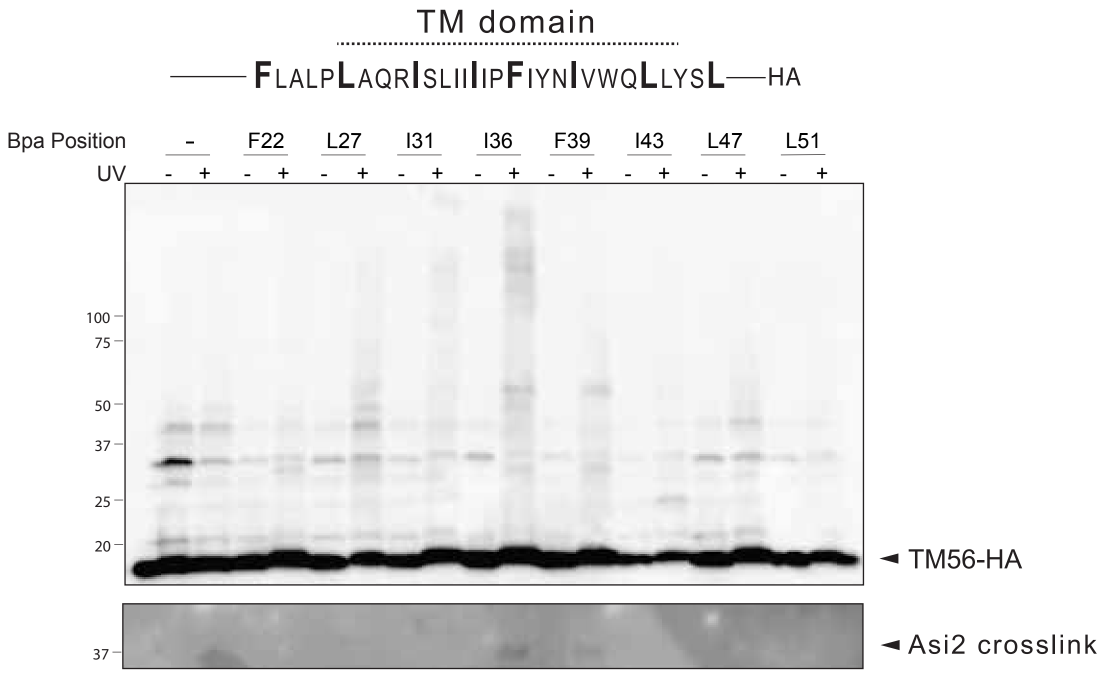
TM domain
FLALPLAQRISLIIIIPFIYNIVWQLLYSL—HA
Bpa Position
-
F22
L27
I31
I36
F39
I43
L47
L51
UV
100
75
50
37
25
20
TM56-HA
37
Asi2 crosslink

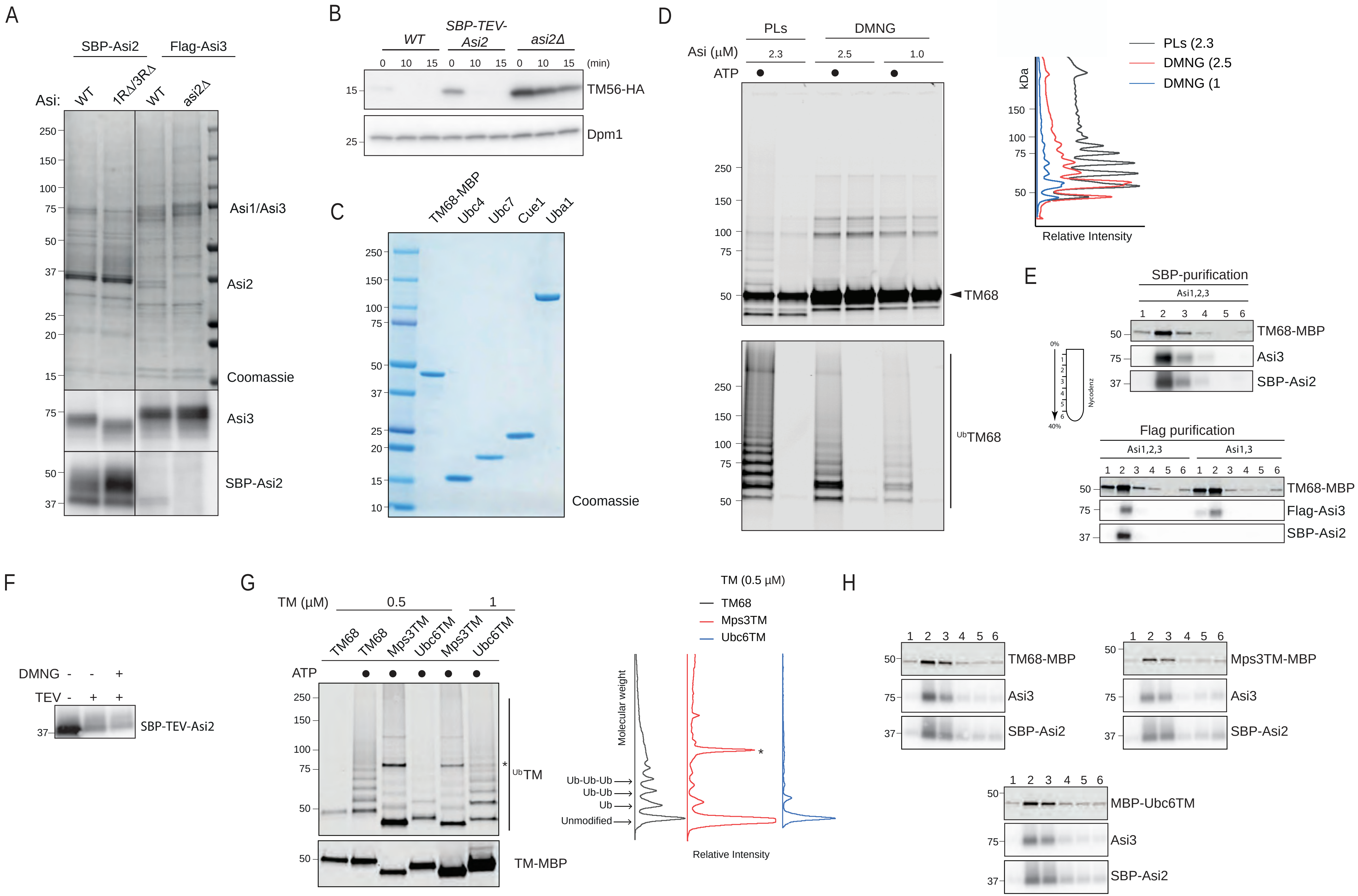

A
SBP-Asi2
Flag-Asi3
Asi:
WT
1RΔ/3RΔ
WT
asi2Δ
Asi1/Asi3
Asi2
Coomassie
Asi3
SBP-Asi2
B
WT
SBP-TEV-Asi2
asi2Δ
(min)
TM56-HA
Dpm1
C
TM68-MBP
Ubc4
Ubc7
Cue1
Uba1
Coomassie
D
PLs
DMNG
Asi (μM)
2.3
2.5
1.0
ATP
TM68
UbTM68
kDa
PLs (2.3
DMNG (2.5
DMNG (1
Relative Intensity
E
SBP-purification
Asi1,2,3
TM68-MBP
Asi3
SBP-Asi2
Nycodenz
Flag purification
Asi1,2,3
Asi1,3
TM68-MBP
Flag-Asi3
SBP-Asi2
F
DMNG
TEV
SBP-TEV-Asi2
G
TM (μM)
0.5
1
TM68
TM68
Mps3TM
Ubc6TM
Mps3TM
Ubc6TM
ATP
UbTM
TM-MBP
TM (0.5 μM)
TM68
Mps3TM
Ubc6TM
Molecular weight
Ub-Ub-Ub
Ub-Ub
Ub
Unmodified
Relative Intensity
H
TM68-MBP
Asi3
SBP-Asi2
Mps3TM-MBP
Asi3
SBP-Asi2
MBP-Ubc6TM
Asi3
SBP-Asi2

A

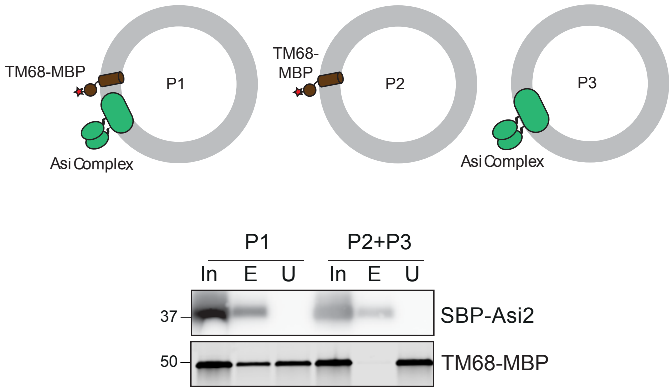
TM68-MBP
P1
TM68-
MBP
P2
P3
Asi Complex
Asi Complex
P1
P2+P3
In
E
U
In
E
U
37
50
SBP-Asi2
TM68-MBP

B

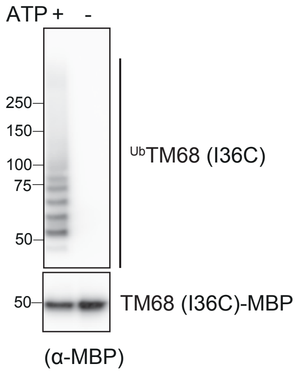
ATP +
-
250
150
100
75
50
UbTM68 (I36C)
50
TM68 (I36C)-MBP
(α-MBP)

C

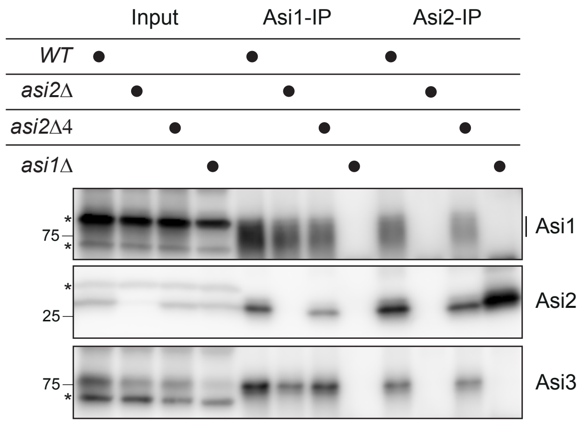
Input
Asi1-IP
Asi2-IP
WT
asi2Δ
asi2Δ4
asi1Δ
75
25
75
Asi1
Asi2
Asi3

Supplemental Table S1, related to STAR methods

PLASMID IDENTIFIER	PRIMERS	PROCEDURE
pPC1230	2015	TM68 (with promoter of Erg11) was obtained by the deletion of amino acids 68-521 by QuickerChange mutagenesis using primer 2015 and pPC1082 was used as as template
pPC1356	2302, 2304	FRB fragment was amplified from plasmid pPC1245 with primers 2302-2304. Fragment digested with SmaI/BamHI and cloned in pPC1354 digested with the same enzymes.
pPC1302	2095, 2096	Insert was amplified from plasmid primers 2095, 2096. Fragment digested with SmaI/BamHI and cloned in pPC1287 digested with the same enzymes.
pPC1313	2182, 2183	Insert was amplified from plasmid primers 2182, 2183. Fragment digested with SmaI/BamHI and cloned in pPC1287 digested with the same enzymes.
pPC1301	2093, 2097	Insert was amplified from plasmid primers 2093, 2097. Fragment digested with SmaI/BamHI and cloned in pPC1287 digested with the same enzymes.
pPC 1229	2014	TM68 (with promoter of Erg11) was obtained by the deletion of amino acids 56-521 by QuickerChange mutagenesis using primer 2014 and pPC1082 was used as template
pESC-Bpa		Chin et al., 2003
pPC1450	2036	Obtained by QuickerChange mutagenesis using primer 2036 was replace F22 by TAG. pPC1229 was used as template
pPC1451	2478, 2479	Obtained by QuickerChange mutagenesis using primer 2478, 2479 to replace L27 by TAG. pPC1229 was used as template
pPC1452	2480, 2481	Obtained by QuickerChange mutagenesis using primer 2480, 2481 to replace I31 by TAG. pPC1229 was used as template
pPC1453	2482, 2483	Obtained by QuickerChange mutagenesis using primer 2482, 2483 to replace I36 by TAG. pPC1229 was used as template
pPC1454	2035	Obtained by QuickerChange mutagenesis using primer 2035 to replace F39 by TAG. pPC1229 was used as template
pPC1455	2484, 2485	Obtained by QuickerChange mutagenesis using primer 2484, 2485 to replace I43 by TAG. pPC1229 was used as template
pPC1456	2486, 2487	Obtained by QuickerChange mutagenesis using primer 2486, 2487 to replace L47 by TAG. pPC1229 was used as template
pPC1457	2488, 2489	Obtained by QuickerChange mutagenesis using primer 2488, 2489 to replace L51 by TAG. pPC1229 was used as template
pPC1082	1557, 185	Foresti et al., 2014
pPC1417	2367, 2368	Asi1 full length amplified using 2367, 2368 primers and inserted into the vector using enzymes SmaI, XhoI
pPC1581	2371, 2372	Codon optimized Asi3 full length amplified using 2371, 2372 primers and inserted into the vector using enzymes SmaI, XhoI
pPC1585	2367, 2759	Asi1 lacking the residues amplified using 2367, 2759 primers and inserted into the vector using enzymes SmaI, XhoI

PLASMID IDENTIFIER	PRIMERS	PROCEDURE
pPC1586	2371, 2760	Asi3 lacking the residues amplified using 2371, 2760 primers and inserted into the vector using enzymes SmaI, XhoI
pPC1583	947,948 (AST lab)	Vector containing an N-terminal SBP-TEV was linearised using primers 947, 948. Asi2 was amplified and inserted into linearised vector by Gibson assembly
pPC1587	2599 , 2600	Obtained by QuickerChange mutagenesis using primer 2599, 2600 to insert the 3xFlag tag. pPC1581 was used as template
pPC1822	2746, 2747, 2744, 2748	Vector containing an H14-Sumo-TM68 was linearised using primers 2746,2747. MBP was amplified using 2744,2748 and inserted into linearised vector by Gibson assembly
pPC1823	2770, 2771, 2765	pPC1822 was linearised using primers 2770, 2771. Gene fragment 2765 containing I36C mutation was inserted by gibson assembly
pPC1555	3363, 3364, 3367	Vector containing H14-Sumo-Mbp was linearised using primers 3363, 3364. Gene fragment 3367 containing Ubc6TM was inserted by gibson assembly
pPC1556	3365, 3366, 3368	pPC1822 was linearised using primers 3365, 3366. Gene fragment 3368 containing Mps3TM was inserted by gibson assembly
pPC 1260	1560+2055	Asi1 fragment was amplified using primers 1560, 2055 and inserted into the vector containing an C-terminal 6xHis tag using enzymes NcoI, XhoI.
pPC1234	1942, 1943	Asi2 fragment was amplified using primers 1942, 1943 and inserted into the vector containing an C-terminal 6xHis tag using enzymes NcoI, XhoI.
pPC 1262	1563, 2056	Asi3 fragment was amplified using primers 1563, 2056 and inserted into the vector containing an C-terminal 6xHis tag using enzymes NcoI, XhoI.
pPC 1878	3334, 3335	Coding region of Ubc4 was amplfied using 3334, 3335 from cDNA of a wild type strain. Amplified product was inserted into the vector by gibson assembly
